# Predictive Removal of Interfacial Defect-Induced Trap States between Titanium Dioxide Nanoparticles via Sub-Monolayer Zirconium Coating

**DOI:** 10.1021/acs.jpcc.2c06927

**Published:** 2022-12-23

**Authors:** Joyashish Debgupta, Leonardo Lari, Mark Isaacs, John Carey, Keith P. McKenna, Vlado K. Lazarov, Victor Chechik, Richard E. Douthwaite

**Affiliations:** † Department of Chemistry, University of York, York YO10 5DD, UK; ‡ Department of Physics, University of York, Heslington, York YO10 5DD, UK; § HarwellXPS, R92 Research Complex at Harwell, Rutherford Appleton Laboratories, Harwell, Didcot OX11 0QS, UK; ∥ Department of Chemistry, University College London, 20 Gordon Street, London WC1H 0AJ, UK

## Abstract

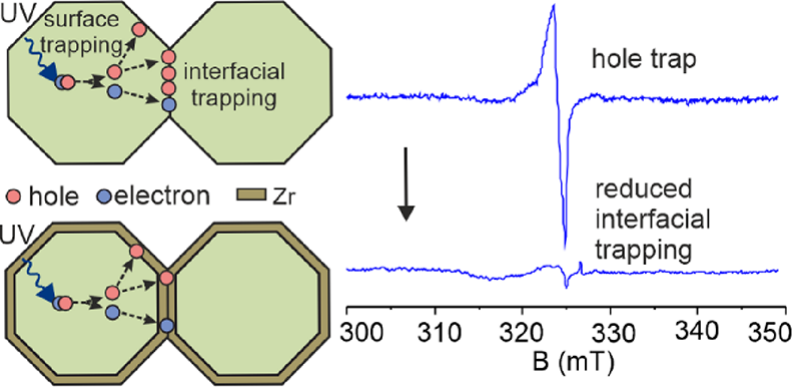

First principles modeling of anatase TiO_2_ surfaces and their interfacial contacts shows that defect-induced trap states within the band gap arise from intrinsic structural distortions, and these can be corrected by modification with Zr(IV) ions. Experimental testing of these predictions has been undertaken using anatase nanocrystals modified with a range of Zr precursors and characterized using structural and spectroscopic methods. Continuous-wave electron paramagnetic resonance (EPR) spectroscopy revealed that under illumination, nanoparticle–nanoparticle interfacial hole trap states dominate, which are significantly reduced after optimizing the Zr doping. Fabrication of nanoporous films of these materials and charge injection using electrochemical methods shows that Zr doping also leads to improved electron conductivity and mobility in these nanocrystalline systems. The simple methodology described here to reduce the concentration of interfacial defects may have wider application to improving the efficiency of systems incorporating metal oxide powders and films including photocatalysts, photovoltaics, fuel cells, and related energy applications.

## Introduction

The low cost, stability, simple processing, and non-toxicity of metal oxide particles have supported their increasing technological application across numerous fields.^1−7^ These include coatings, catalysis, and energy conversion, providing impetus for further study particularly of nano-sized powders and nanoporous film morphologies formed from nanoparticle sintering.^8^ The high surface area-to-volume ratio of nanoparticles renders surface and interfacial phenomena particularly prevalent. A key phenomenon is the nature and role of surface and interfacial defects in semiconductor oxides, which are pervasive at the nanoscale and depend on the synthetic and processing procedures during production.^9−11^ The role of surface and interfacial defects can be complex, but they are often considered detrimental to charge carrier mobility and the performance of, for example, photocatalysts and photoelectrochemical cells by trapping photo-initiated charge carriers and increasing electron–hole recombination.^12^ Other important applications of defect containing nanoporous metal oxide films include electron transport layers in solar cells and electrodes in fuel cells, where good charge carrier mobility is also an essential requirement.^13−15^

The trapping of charge carriers at interfacial defects is thought to be the primary reason for reduced electron and hole mobility compared to single crystals. For example, the mobilities of porous TiO_2_, ZnO, and SnO_2_ films have been shown to be between two and four orders of magnitude smaller than those of the corresponding single crystals.^16^ Unfortunately, notwithstanding their wide application, the charge mobility in nanoporous metal oxide films is generally poorly understood. Strategies to mitigate surface and interfacial defects are mainly empirically driven. Progress has been limited, and little is known, quantitatively, about the atomistic origin of charge trapping, which presents an obstacle to knowledge-led optimization of wide-ranging applications.

**Figure 1:**
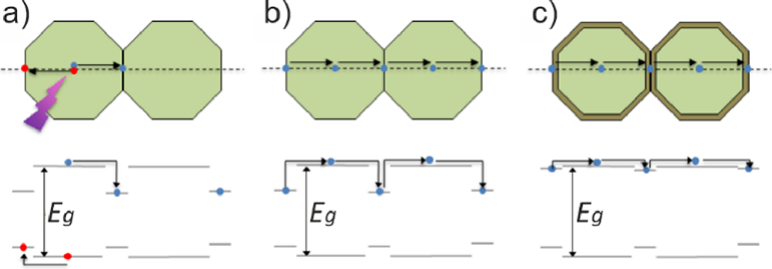
(a) An illustration of possible charge trapping processes on photoexcitation of nanocrystalline TiO_2_. Photoexcited electrons and holes can become trapped at surfaces and interfaces (a corresponding energy level diagram for this process is also shown below). Here, the electron and hole are separated but if the electron and hole localize near the same defect, it can promote recombination. (b) An illustration of how electron trapping at interfaces and surfaces in *n*-type TiO_2_ can reduce long-range electron mobility by several orders of magnitude. (c) Suitable chemical modification of the nanoparticles to remove or reduce the depth of such traps should improve the long-range mobility.

In general, many defect states are introduced into the bandgap of a semiconductor via distorted atomic arrangements compared to the idealized bulk, and clearly surface termination will drive significant distortion and possible introduction of deep-lying states with the potential to trap photoexcited electrons and holes and promote charge separation and/or recombination (Figure 1a) as well as reduce charge carrier mobility (Figure 1b). In principle, chemical modification with the introduction of ions can induce structural and electronic modifications to eliminate these deep-lying states (Figure 1c). Such modifications have been attempted previously but due to the lack of understanding on the origin of charge trapping or the effects of surface modification success has been limited.^17−19^

The subject of this study, anatase TiO_2_, is a prototypical metal oxide with a multitude of applications dependent on charge mobility including solar cells, photocatalysis, and batteries.^20−25^ Both in and out of equilibrium (e.g., following photoexcitation or during non-equilibrium charge transport), the trapping of electrons and holes by defects plays a critical role in determining its (opto)electronic and photochemical properties. Defects in this respect include intrinsic point defects such as vacancies and interstitials, extrinsic impurity defects, surfaces (which themselves may include varied topological features such as steps, kinks, edges, and corners), and interparticle interfaces. A number of experimental techniques have been be used to probe charge trapping phenomena in anatase, including electron paramagnetic resonance (EPR) spectroscopy,^26,27^ photoluminescence spectroscopy,^28^ and scanning tunneling microscopy.^29^ However, a definitive understanding of charge trapping behavior has yet to emerge, in part as it is very challenging to disentangle the effects of the many different defects present in porous nanocrystalline films, as well as the fact there is significant variability in material properties depending on the synthesis procedure. There is however clear evidence for hole trapping in anatase^27^ as well as a band of electron trapping states below the conduction band minimum that has variously been attributed to surfaces, oxygen vacancies, titanium interstitials and hydroxyl groups.^30,31^

To address this important problem, there have been numerous first principles theoretical studies of charge trapping in TiO_2_ (usually using density functional theory) especially for the two most common polymorphs: rutile and anatase.^32−34^ Unfortunately, the picture with respect to charge trapping remains somewhat unclear due to numerous conflicting predictions depending on the approximations used (e.g., exchange-correlation approximation and supercell size). To be able to predictively model charge trapping at a range of defects in porous nanocrystalline films (some of which require very large supercells), it is important to employ both an efficient and carefully parameterized approach. We recently tackled this challenge by combining an efficient hybrid functional approach implemented in the CP2K code with an ab initio determination of the proportion of Hartree–Fock exchange.^35^

Subsequently, using this approach we have investigated a range of charge trapping defects and explored chemical modifications to reduce charge trapping and improve mobility. Herein, we describe a theory-led study of a chemical modification of TiO_2_ nanoparticles to reduce charge carrier trapping associated with surface and interfaces. Nanoparticle morphologies of anatase TiO_2_ have been prepared, characterized, and guided by theoretical predictions, subsequently chemically modified. Comparison of structural and spectroscopic data of unmodified and modified TiO_2_ nanoparticles and nanoporous film morphologies shows that Zr doping reduces the concentration of hole trap states on photoexcitation. Furthermore, conductivity and mobility of nanoporous films measured using electrochemical impedance spectroscopy indicate increased electron conductivity and mobility after Zr modification; however, other grain boundary factors dominate the reduced conductivity of nanoporous films compared to single crystals.

## Methods

### Density Functional Theory Calculations

We employ density functional theory with a hybrid functional approximation for exchange and correlation to model charge trapping in TiO_2_.^32^ To minimize self-interaction errors and thereby ensure accurate predictions of carrier localization, we parameterize the proportion of Hartree–Fock (HF) exchange in the functional to ensure linearity of the total energy with respect to electron occupation number (a known property of an exact functional). This approach gives excellent results for electron densities and energies when compared to exact many body solutions for model one-dimensional systems^35^ and for various TiO_2_ polymorphs gives results consistent with experiment. For anatase TiO_2_ (the focus of this study), the optimized HF proportion is 10.5% and we predict that electrons do not self-trap in the bulk crystal to form a small polaron but holes do (with a trapping energy of −0.2 eV and an unoccupied electronic state around 1 eV above the valance band maximum). These predictions are consistent with the high mobility of anatase single crystals as well as EPR and photoluminescence spectroscopy data.^26−28,36^ Full details of the approach including basis sets, supercell size, and parameterization of the proportion of HF exchange for different TiO_2_ polymorphs are provided in ref (32) and boundary structures for particle interfaces in refs (43, 47).

### Synthesis of TiO_2_ Nanoparticles Stabilized with Surface Ligands (SL-TiO_2_)

The solvothermal method described by Dinh et al.^37^ was used for the synthesis of rhombic-shaped TiO_2_ nanoparticles stabilized by a mixture of surface ligands oleic acid (OA) and oleylamine (OAM). Briefly, titanium tetrabutoxide (1.78 g, 5 mmol), OA (7.18 g, 20 mmol), and OAM (6.76 g, 30 mmol) were mixed in EtOH (5.8 mL, 100 mmol) in a 40 mL Teflon cup with stirring. The cup containing the reaction mixture was transferred into a 100 mL Teflon-lined stainless steel pressure reactor containing 96 v/v% ethanol in deionized water (20 mL). The system was then heated at 180 °C for 18 h to give a white precipitate of SL-TiO_2_ that was washed with ethanol twice and dried in a vacuum oven at room temperature overnight.

### Removal of Surface Ligands from SL-TiO_2_ Nanoparticles (TiO_2_)

SL-TiO_2_ (868 mg) was dissolved in hexane (25 mL), and the suspension was mixed with NOBF_4_ (1.39 mg, 12 mmol) in acetonitrile (25 mL). The resulting mixture was sonicated until the off-white color of the mixture became bright white and the TiO_2_ nanoparticles accumulated in the lower acetonitrile layer and separated by centrifugation at 4400 rpm for 10 min. The nanoparticles were then re-dispersed in dimethylformamide (20 mL) and flocculated using toluene (ca. 20 mL) and isolated using centrifugation. This procedure was repeated twice more, and finally the particles of TiO_2_ were stored in dimethylformamide (20 mL) to prevent agglomeration. TiO_2_ was recovered from the dimethylformamide suspension by centrifugation after adding excess toluene and used as required.

### Zirconium Modification of TiO_2_ (Zr-TiO_2_)

TiO_2_ (120 mg) was suspended in ethanol (50 mL) containing H_2_ZrF_6_ (0.02, 1.00, or 5.00 mM) and stirred overnight at room temperature. The modified particles were collected by centrifugation at 4400 rpm for 10 min and then dried in a vacuum oven at room temperature without further washing to give Zr-TiO_2_. H_2_ZrF_6_-modified TiO_2_ was also prepared from an aqueous solution of H_2_ZrF_6_ using the same method. Similarly, ZrCl_4_ 2H_2_O, ZrO_2_Cl_2_*x*H_2_O, and ZrO(NO_3_)_2_*x*H_2_O were used as other zirconium precursors to modify TiO_2_. The method and solvent used was identical to that applied for H_2_ZrF_6_ except for ZrCl_4_ 2H_2_O, which was soluble in dry THF.

### Acid Modification of TiO_2_

TiO_2_ (120 mg) was suspended in either 1 M HOAc (50 mL) or 2 mM HNO_3_ ethanolic solution (50 mL) and stirred overnight at room temperature. The particles were collected by centrifugation at 4400 rpm for 10 min and dried in a vacuum oven for 12 h at room temperature without further washing.

### Preparation of Films for Electrochemical Measurements

TiO_2_ or Zr-TiO_2_ (120 mg) was dried under vacuum at 40 °C overnight in a vacuum oven and ground using an agate mortar and pestle several times using a few drops of EtOH to lubricate grinding. A 3:2 EtOH:H_2_O solution (200 μL) was added and ground further to obtain a slightly viscous slurry, and 10 μL of this slurry was put on a substrate (clean FTO), which was modified by the addition to two strips of Scotch tape (∼62 μm thick) covering 3 mm of the outer edges and immediately spread with a clean glass slide. After air drying, the films were sintered at 450 or 550 °C for 1 h at a heating ramp of 10 °C min^–1^ in air. Films were stable after sintering with no sign of delamination during handling.

### Characterization Methods

Carbon, hydrogen, and nitrogen (CHN) analysis was performed using an Exeter Analytical Inc. CE-440 analyzer in conjunction with a Sartorius SE2 analytical balance. ICP-MS was undertaken at the University of Hull. Infrared spectra were recorded on a PerkinElmer FT-IR Spectrum Two spectrometer in the region of 4000–500 cm^–1^ collected over eight scans with a resolution of 1 cm^–1^. ATR-IR spectra were collected by first cleaning the crystal with isopropanol and measuring a blank spectrum to use as reference. Powder x-ray diffraction (PXRD) data was collected using a Bruker D8 powder diffractometer equipped with a Cu Kα source with a 0.02° step size. Brunauer–Emmett–Teller (BET) surface area measurements were collected using a Micromeritics ASAP 2020 Porosimeter. 30 mg of sample was heated to 150 °C for 4 h under nitrogen flow to degas and dry sample prior to weighing in triplicate. BET (N2) surface area analysis was performed on a five-point pressure range. Scanning electron microscopy (SEM) imaging and elemental analysis were carried out on a JEOL 7800F field emission microscope equipped with two Oxford Ultim Max EDX detectors and Aztec analysis software. High-resolution transmission electron microscopy images were acquired using a double aberration-corrected 200 keV JEOL 2200FS (scanning)/transmission electron microscope ((S)TEM) with a field emission gun equipped with a Thermo Scientific Noran 7 energy-dispersive X-ray (EDX) detection system. Sample preparation for TEM analysis was done by dusting sample powder following a literature procedure^38^ onto holey carbon films supported by 300 mesh TEM Cu grids (Agar Scientific, S147-3) thermally pre-treated.^39^ XPS measurements were taken using a Kratos Axis Supra using a monochromated Al-ray anode (1486.69 eV) and fitted with a charge neutralizer. All measurements were performed with a pressure of <10^–8^ Torr. Wide scans were performed using a pass energy of 160 and step size of 1 eV and high-resolution scans with a pass energy of 20 and step size of 0.1 eV. Data was analyzed using CASAXPS v2.3.15. O 1s peaks were fitted using a LA(1.53, 253) line shape. Ti 2p peaks were fit with varying FWHM, allowing for broadening effects due to Coster–Kronig transitions, and a doublet separation of 5.7 eV. Zr 3d speaks were fit with a doublet of 2.4 eV. All spectra were charge corrected to adventitious carbon at 284.8 eV. UPS measurements were recorded using a He(I) lamp attached to the chamber of the Supra, permitting analysis of the same spot as for XPS. Measurements were recorded with an emission current of 20 mA, and a pass energy of 10 eV. X-band continuous-wave electron paramagnetic resonance (CW-EPR) spectra were recorded on a JEOL JES-RE1X EPR spectrometer equipped with a cylindrical cavity. A modulation frequency of 100 kHz, modulation amplitude of 0.079 mT, sweep width of 25 mT, time constant of 0.03 s, and microwave power of 5 mW were generally used. Spectra were recorded in vacuum (10^–4^ to 10^–5^ mbar) at 77 K unless stated otherwise. All spectra were recorded using 100 mg of sample. Spectra are an average of five consecutive scans to improve signal to noise. The *g*-values were determined using a DPPH standard. A finger Dewar filled with liquid N_2_ was used to cool samples, which was continuously purged with He gas to reduce boiling. Samples (100 mg) were sintered inside the EPR tube and ex situ of the spectrometer using a homemade heating assembly controlled by a digital heating controller. Samples were illuminated in situ using a 100 W high-pressure mercury lamp with a 20 min pre-illumination period before EPR spectra were recorded. All electrochemical measurements were carried out in a homemade three-electrode electrochemical cell using the films as the working electrode, Pt wire as the counter electrode, and Ag/AgCl (3 M NaCl) as the reference electrode at room temperature. The electrolyte was purged with N_2_ for 30 min prior to every voltametric experiment. A BioLogic SP-150 potentiostat controlled by EC-Lab (V11.20) software was used to perform all the measurements. Cyclic voltammetry experiments were initiated at the most positive potential and swept toward a more negative value at a scan rate of 10 mVs^–1^ (unless otherwise specified). Electrochemical impedance spectroscopy (EIS) data were recorded from 10 kHz to 10 mHz at an amplitude of 5 mV with 10 points per decade at open circuit potential (OCP) and at various DC potentials (vs Ag/AgCl, 3 M NaCl). All EIS experiments were carried out using air saturated electrolyte. EIS data analysis including Mott–Schottky analysis was performed using EC-Lab software.

## Results

### Theoretical Screening of Potential Dopants to Reduce Surface and Interfacial Charge Trapping

Our previous theoretical calculations using density functional theory have provided atomistic insight into charge trapping in nanoporous anatase TiO_2_ films and identified prospective chemical modifications to reduce charge trapping and improve mobility.^32,35,40−44^ These results and new calculations (see below) have been used to guide the experimental work described herein.

**Figure 2:**
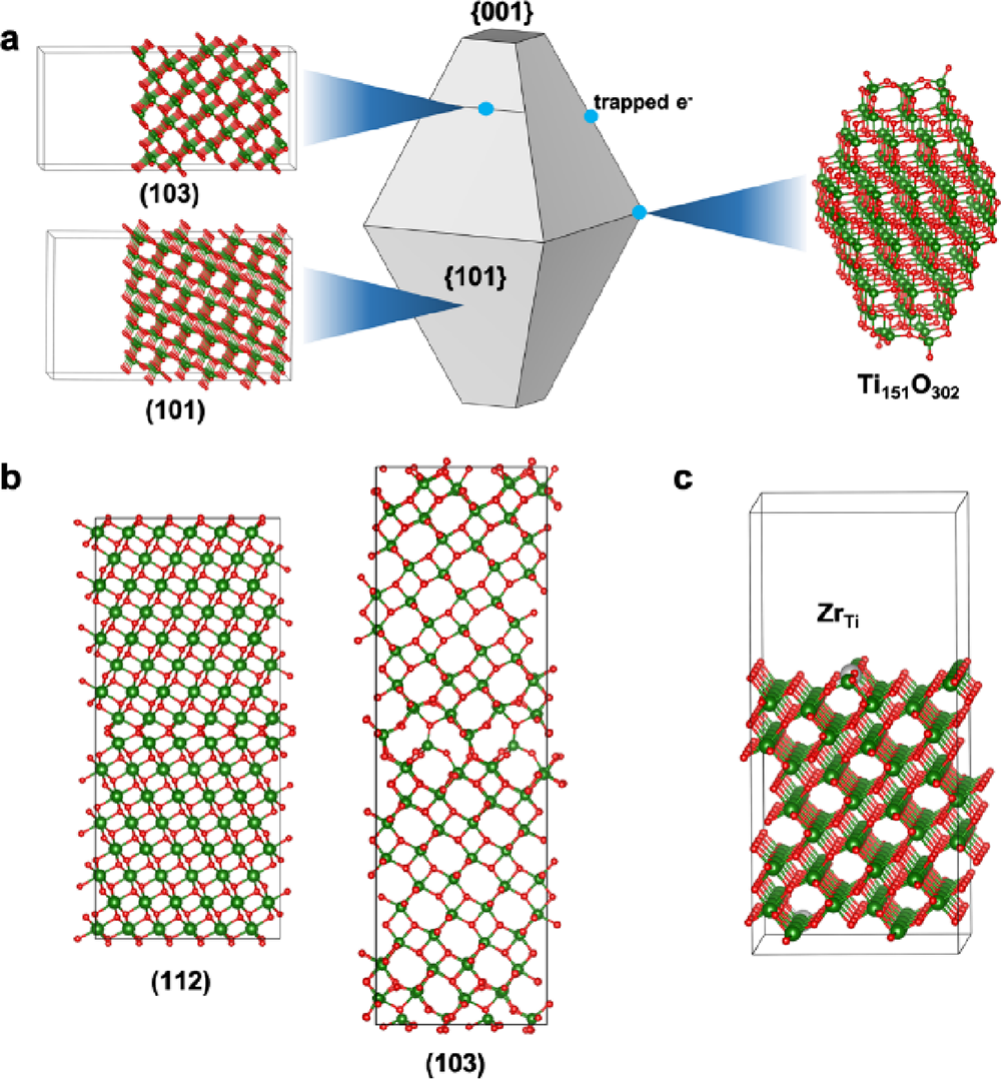
(a) The predicted equilibrium crystal shape of anatase TiO_2_ exposing primarily {101} and {001} facets. Surface slab models for the (101) and (103) surfaces as well as a model of a finite nanoparticle are also shown. Blue circles indicate the sites predicted to trap electrons, which include steps, edges between facets, and corners. (b) Supercell models for the Σ3 (112) twin boundary and (103) grain boundary. (c) Substitution of undercoordinated surface Ti atoms with Zr, which is predicted to be a plausible strategy to reduce electron trapping and improve mobility. Titanium atoms are represented by green spheres and oxygen atoms by red spheres.

Previous work has shown that one can model the structure, stability, and charge trapping properties of extended surfaces of anatase TiO_2_ using the periodic slab approach. By considering all low index surface terminations, the equilibrium crystal shape for anatase TiO_2_ nanocrystals is obtained via the Wulff construction (Figure 2a). These low index surfaces are predicted to trap holes in the bulk and more strongly at surfaces, by up to 1 eV, whereas electrons do not trap.^40,41^

Of course, real nanoparticles are unlikely to be perfectly faceted in this way and one should expect steps and other topological defects on the surface. Higher-index stepped surfaces can be used as a simplified model for such defects as indicated schematically in Figure 2a (left), but lower-symmetry topological defects such as edges and corners are more challenging to model using extended surfaces. Alternatively, a finite (albeit small compared to experiment) nanoparticle as shown in Figure 2a (right) reveals electron trapping at a wide range of undercoordinated surface Ti sites, giving rise to a band of states 0.5–1.0 eV below the conduction band minimum.^42^

Similarly, interfaces between particles can be modeled (Figure 2b), and consistent with the results for low index surfaces, the experimentally observed high symmetry Σ3 {112} twin boundary^45,46^ is predicted to weakly trap holes by 0.15 eV, but not electrons.^43^ More recently, lower-symmetry {310} and {103} grain boundary defects in anatase have been predicted to trap electrons, which was confirmed by electron energy loss spectroscopy measurements.^47^

**Figure 3:**
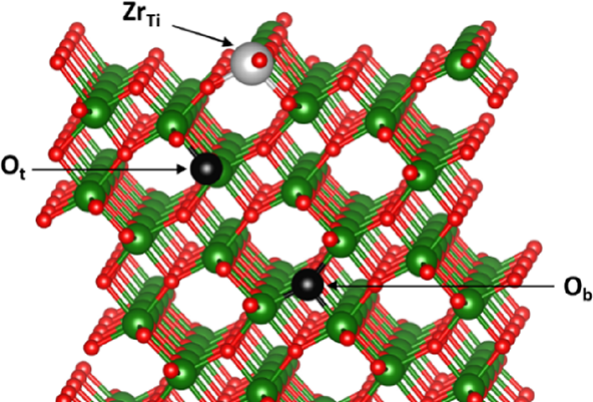
The {103} anatase TiO_2_ surface modified with Zr (a single surface Ti is substituted for Zr). The most favorable oxygen site for trapping a small hole polaron is labeled as O_t_, while a bulk-like O site in the center of the surface slab model is labeled O_b_. Titanium atoms are represented by green spheres and oxygen atoms by red spheres.

In summary, low index surfaces and interfaces do not have a strong tendency to trap electrons but are favorable locations for hole trapping, whereas undercoordinated and/or strained Ti ions near steps, edges, corners, and lower symmetry interfaces are predicted to trap electrons. These results indicate that the charge trapping properties of surface and interface defects are quite similar, suggesting that surfaces may be used as simple models for interfaces. Subsequently, we performed a systematic screening of possible chemical modifications to the {103} surface. This surface exposes low coordinated atoms at the surface steps and thus should provide a more representative model of more general nanoparticle surfaces and interfaces to identify strategies to eliminate the deep electron traps, which limit the mobility of *n*-type anatase TiO_2_.^44^ Two approaches were considered: surface cation substitution (V, Sb, Sn, Zr, and Hf) and alkali metal doping (Li, Na, K, Rb, and Cs). We found that Zr and Hf are effective in preventing electron trapping (Figure 2c) and that alkali metal doping is also predicted to improve electron mobility via the donation of electrons to fill deep trap states. Consideration of possible experimental approaches to test these predictions (see below) identified Zr substitution as a plausible strategy. We have therefore again used analogous first principles calculations to explore the possibility that Zr modification of surface and interfaces can reduce hole trapping on the {103} surface. For the unmodified surface, we predict that holes can trap to form small polarons and the most stable site for hole trapping is in the sub-surface region with a trapping energy of −90 meV with respect to a small hole polaron localized on a bulk-like O site (see sites labeled O_t_ and O_b_ in Figure 3). On Zr modification of the surface, the trapping energy is reduced significantly (to −4 meV), indicating a reduced tendency for holes to trap in the surface region.

Based on these predictions, we focused on the chemical modification of TiO_2_ nanoparticles via Zr substitution to assess the impact on charge trapping and mobility.

### Synthesis, Composition, and Structural Characterization of TiO_2_ and Zr-TiO_2_

**Figure 4:**
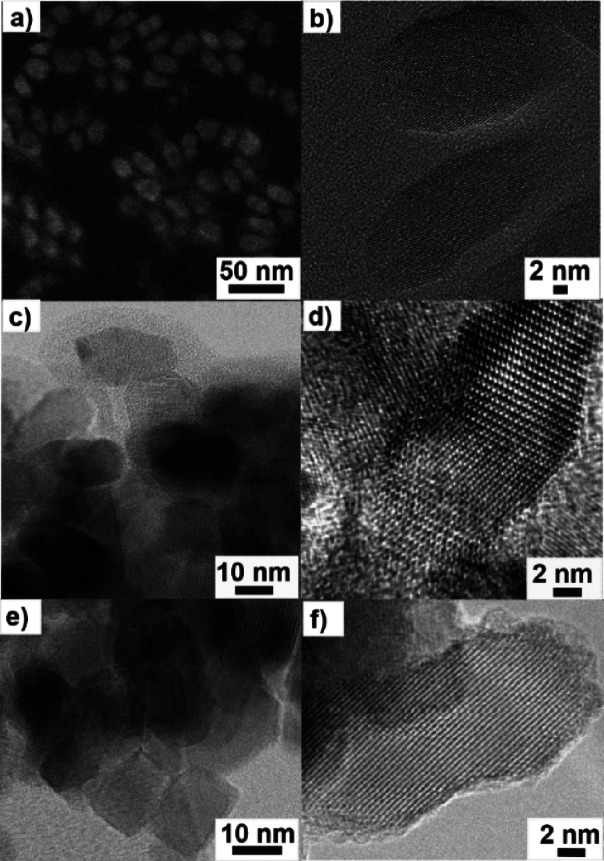
(a) SEM of SL-TiO_2_, (b) TEM of SL-TiO_2_ nanoparticles, (c, d) TEM of TiO_2_ sintered at 550 °C in air, and (e, f) TEM of Zr-TiO_2_ (1.00 at%) sintered at 550 °C in air.

To restrict the number of surface structures and range of interfacial interactions, crystalline TiO_2_ nanoparticles of a single polymorph with narrow size dispersity were targeted. In addition, to increase the real-world relevance, no specialist precautions were undertaken to prevent ambient surface contamination during synthesis and processing of the nanoparticles. Anatase TiO_2_ nanoparticles were prepared using a reported procedure^37^ via hydrolysis of titanium(IV) butoxide in the presence of surface binding ligands of OA and OAM to give SL-TiO_2_ (where SL denotes the presence of surface ligands). Surface ligands were then removed by treatment with NOBF_4_ to give TiO_2_. FT-IR spectroscopy (Figure S1) of TiO_2_ indicates removal of organic moieties and the absence of [BF_4_]^−^ on the surface. CHN elemental analysis (Table S1) gives ∼2–3 wt % C and 0.4 wt % H attributable to adsorbed DMF, which is observed in the IR spectrum (Figure S1). SEM (Figure 4a) and (S)TEM) (Figure 4b,c) of SL-TiO_2_ and TiO_2_ show an average particle size of ca. 15–20 nm and rhombic morphology with {101} predominant facets (Figure S2a-c), which is consistent with reported theoretical predictions.^40^ Selected area electron diffraction (SAED) (Figure S2d-e) shows reflections consistent with single crystalline particles of the anatase polymorph, and bulk phase purity was confirmed using PXRD (Figure S3). The surface area determined from a N_2_ adsorption isotherm is 107 m^2^g^–1^, which on sintering at 550 °C reduces to 63 m^2^g^–1^ (Figure S4). TEM analysis of sintered TiO_2_ shows that particle growth does not occur but new particle–particle interfaces are formed (Figure 4c,d).

Practically, it is very challenging to obtain selective and quantitative surface modifications corresponding to a theoretical predictive model. Our simpler strategy was to disperse TiO_2_ nanoparticles in a solution containing a soluble Zr precursor with, ideally, molecular speciation, which can adsorb onto the surface of TiO_2_, thus limiting surface coverage to a sub-monolayer. Subsequent heating would drive surface and interfacial structure changes commensurate with the predicted thermodynamically stable structures leading to a reduction in the number of observable trap states.

Surface doping of TiO_2_ nanoparticles with Zr was undertaken after surface ligand removal and before any subsequent thermal processing. Various solvents and Zr precursors (ESI) were screened at a range of concentrations to obtain stable dispersions of TiO_2_ nanoparticles and a Zr precursor. Dispersions were stirred for 12 h, and the Zr-modified TiO_2_ powder (Zr-TiO_2_) isolated via centrifugation. This powder was then either sintered or re-dispersed and used to form nanoporous films using a doctor blade method, which were also then subsequently sintered. To optimize surface doping with Zr, the trap state speciation and concentration were semi-quantitatively screened using EPR spectroscopy, as described below. Ultimately, an ethanolic dispersion of TiO_2_ nanoparticles containing H_2_ZrF_6_, sintered at 550 °C, gave the greatest reduction in surface and interfacial trap states.

Three concentrations of H_2_ZrF_6_ from micromolar to millimolar were used to target sub-monolayer coverage of TiO_2_ nanoparticles based on estimates of the surface:bulk ratio of Ti atoms (ESI). Inductively-coupled plasma mass spectrometry (ICP-MS) gave Zr concentrations of 0.12, 1.00, and 3.15 at %, respectively (Table S2). Using 1.00 at% as an example, if it is assumed that all of the Zr atoms are present at the surface, a coverage of 0.7 Zr atoms per nanometer squared is estimated (ESI) indicating sub-monolayer coverage. Further analysis by TEM (Figure 4e,f) and N_2_ adsorption surface area determination (Figure S4) show that sintered (550 °C) powders of Zr-TiO_2_ exhibit no morphological or textural differences when compared to TiO_2_. PXRD data are also essentially identical (Figure S3). STEM analysis with elemental mapping using energy-dispersive x-ray (EDX) analysis (Figure S5) shows no evidence of Zr clustering.

**Figure 5:**
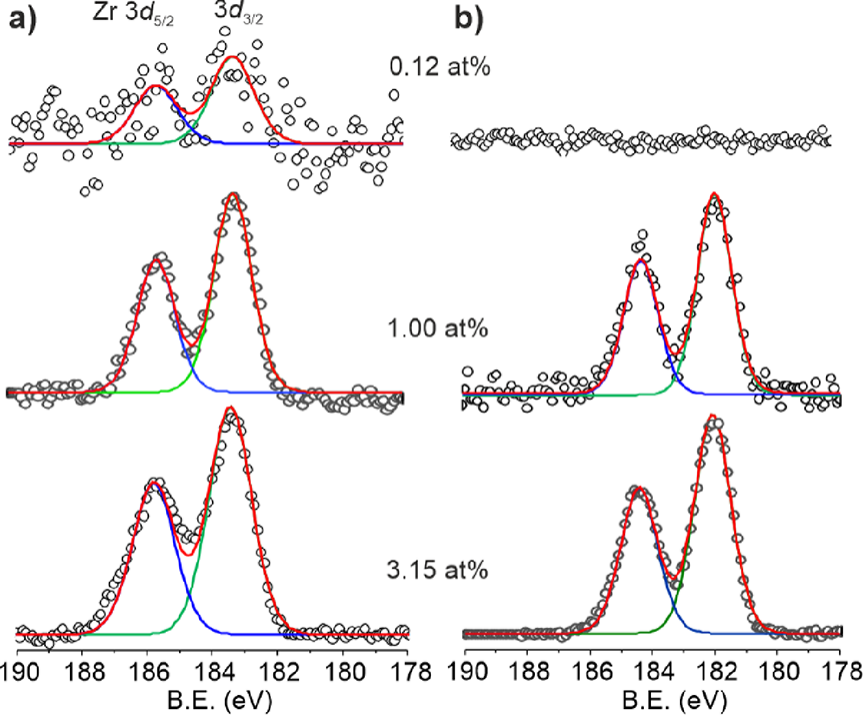
Zr 3d X-ray photoelectron spectra of (a) unsintered and (b) sintered (550 °C) Zr-TiO_2_ (with 0.12, 1.00, and 3.15 at% respectively. Open circle (o) raw data, red line (—) overall fit, green (—), and blue (—) lines are peak fits, respectively.

X-ray photoelectron spectroscopy (XPS) was performed before and after sintering at 550 °C of TiO_2_ and a Zr-TiO_2_ series with Zr concentrations of 0.12, 1.00, and 3.15 at %, respectively. Ti 2p spectra were indistinguishable for all samples (Figure S6) showing peaks with a binding energy (BE) at 458.6 and 464.2 eV corresponding to Ti 2p_3/2_ and Ti 2p_1/2_, which is similar to reported data for TiO_2_,^48^ indicating no significant differences in the chemical environment of Ti(IV) ions or detectable quantities of Ti(III).^49,150^ O 1s spectra (Figure S7) are also indistinguishable from each other and that reported for TiO_2_.^48,49^ Before sintering, Zr 3d spectra showed two peaks at 183.1 and 185.4 eV attributable to Zr3d_5/2_ and Zr3d_3/2_ for the three Zr-containing samples, albeit weakly for the lowest concentration (Figure 5a). These data indicate that the speciation of the adsorbed Zr derived from H_2_ZrF_6_ is the same for each concentration. Furthermore, the BE is greater than for, e.g., ZrO_2_, consistent with fluoride coordination.^50^ After sintering at 550 °C, the Zr 3d spectra exhibit clear concentration-dependent differences indicative of new speciation (Figure 5b). The highest concentration (3.15 at%) shows peaks at 183.4 and 185.8 eV, which are similar to reported data for ZrO_2_,^51,52^ whereas 1.00 at% exhibits lower BE peaks at 181.9 and 184.3 eV. The lower BE observed for sintered Zr-TiO_2_ (1.00 at%) is consistent with a more electron-rich environment for the Zr(IV) ions compared to ZrO_2_ consistent with a lower coordination number of surface bound Zr ions as envisaged by the theoretical predictions. Furthermore, compositional analysis by XPS (Table S3) after sintering shows that the Zr concentration decreases for all sintered Zr-TiO_2_, indicating some diffusion of Zr below the surface. For the lowest concentration (0.12 at%), the Zr content is below the detection limit after sintering (Figure 5b).

### Electronic Properties and Charge Trapping Comparison of TiO_2_ and Zr-TiO_2_

Ultraviolet photoelectron spectroscopy (UPS) was used to probe the valence band and surface electronic properties of TiO_2_ and Zr-TiO_2_ after sintering at 550 °C. For sintered TiO_2_ (Figure S8), the valance band edge (E_VB_) is 2.6 eV vs Au (−7.7 eV vs vacuum) with an average work function (ϕ) of 6.6 eV. These data are consistent with studies of various polycrystalline anatase morphologies and single crystal surfaces.^53^ For example, E_VB_ = 2.5 eV and ϕ = 6.5 eV for the (001) surface of anatase single crystals.^54^ Sintered Zr-TiO_2_ exhibit greater E_VB_ and ϕ (−7.9 and 6.6 eV (Zr = 0.12 at%), −8.2 and 6.8 (1.00 at%), and – 8.2 and 7.1 (3.15 at %). In comparison, ZrO_2_ has reported E_VB_ = ca. −8.4 eV.^55,56^ Reported work function data for ZrO_2_ varies widely, which limits confident comparison;^57^ however, the data here are within range. Collectively, these data are consistent with the surface more closely approximating that of ZrO_*x*_ as the Zr concentration increases. In addition, the low energy region of sintered Zr-TiO_2_ exhibits new peaks in comparison to sintered TiO_2_ (Figure S8), reflective of changes to surface states; however, UP spectra of polycrystalline materials contain contributions from many surfaces preventing unequivocal assignment. Nevertheless, previous experimental and theoretical work on single crystal surfaces of TiO_2_ have attributed peaks at 11 eV to surface OH groups, which is present for TiO_2_ and all Zr-TiO_2_.^56,58^ Below, 10 eV peaks are attributed to bonding and non-bonding states of O 2p and those hybridized with Ti 3d orbitals, which are clearly modified on the addition of Zr to TiO_2_ reflecting surface changes.

**Figure 6:**
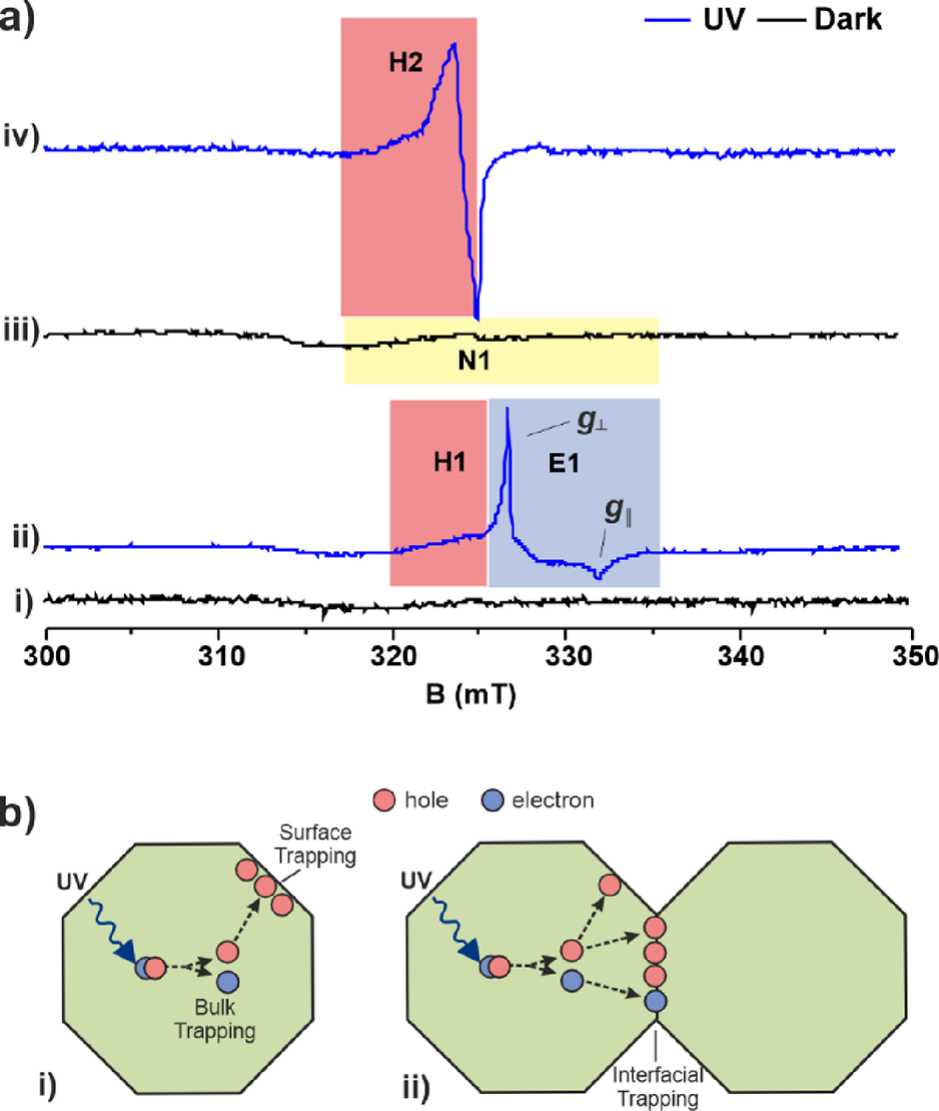
(a) EPR spectra of TiO_2_. (i) in the dark before sintering; (ii) under continuous UV illumination before sintering; (iii) in the dark after sintering at 550 °C; (iv) under continuous UV illumination after sintering at 550 °C. All spectra were recorded at 77 K under vacuum (<10^–4^ mbar). (b) (i) Proposed processes on illumination before sintering. Photogenerated electrons in TiO_2_ are trapped by bulk Ti^4+^ to form Ti^3+^ (E1), whereas the holes migrate and are trapped by surface OH^–^ sites (H1). (ii) Proposed processes on illumination after sintering at 550 °C. The photogenerated holes are mostly trapped at the interfacial sites (H2), whereas some of the photogenerated electrons are either partially trapped at the interface or react with trapped NO (loss of N1 signal).

EPR spectroscopy of TiO_2_ and Zr-TiO_2_, both before and after sintering, was used as a simple semi-quantitative measure to assess the evolution of trap states as a function of Zr concentration and processing parameters. In addition, EPR was undertaken in the dark and under UV illumination to study the consequence of Zr doping on electron and hole trapping of TiO_2_ relevant to the use of TiO_2_ as an electron transport layer, and as a photocatalyst under illumination. EPR spectra of TiO_2_ before sintering in the dark (Figure 6a(i)) is almost featureless with occasional appearance of a broad symmetric signal. Continuous illumination with UV light shows a sharp axial signal, E1 consisting of two components, E1(*g*_⊥_) = 1.992 and E1(*g*_∥_) = 1.962, attributed to trapped electrons at bulk Ti^3+^ sites in anatase, which are in agreement with literature reports (Figure 6a(ii)).^26^ The spectrum also contains a broad signal, H1 with *g* value ranging from 2.0061 to 2.0117, which is consistent with oxygen-centered radicals reported in the range from 2.00 to 2.03 for surface hole traps of hydrated TiO_2_-containing OH^–^ groups. For example, photogenerated holes are trapped at the lattice oxygen atoms located in the subsurface layer of hydrated anatase particles attributed to Ti^4+^O^2–^Ti^4+^OH^–^.^59^

In comparison, the dark spectrum of sintered TiO_2_ (Figure 6a(iii)) consists of a rhombic signal N1 *g*_1_ = 2.0037, *g*_2_ = 1.9997, *g*_3_ = 1.92635 with hyperfine splitting constants of 0.71 (A1), 33 (A2), and 14 G (A3), respectively. A similar set of dark signals is observed when SL-TiO_2_ is directly sintered in air without using NOBF_4_ treatment for surface ligand removal (Figure S9). Spectral simulation (Figure S10) and comparison with literature data^60,61^ are consistent with *N*-doping of TiO_2_ resulting in trapped NO molecules in TiO_2_ micro voids, which are surface bound at 77 K. Continuous UV illumination of sintered TiO_2_ at 77 K generates signal H2 with *g* values ranging from 2.032 to 2.002 (Figure 6a(iv)), which on warming to room temperature in the dark reverts back to the dark signal (Figure 6a(iii)). Simulation (Figure S11) suggests that there are at least three components to the spectrum and based on the simulated *g*-values and the quenching experiments described below, the data are consistent with trapped holes associated with Ti-dioxo, -oxo, and -hydroxy containing species.^59^ To gain more insight into the nature and location of the photogenerated H2 signal, electron and hole quenching experiments were performed using O_2_ and 2-propanol vapor, respectively (Figures S12 and S13). The H2 signal is unaffected by the presence of O_2_ or 2-propanol vapor, indicating that the trapped holes of H2 are located at the interfacial or subsurface region and are not accessible to external probe molecules.

**Figure 7:**
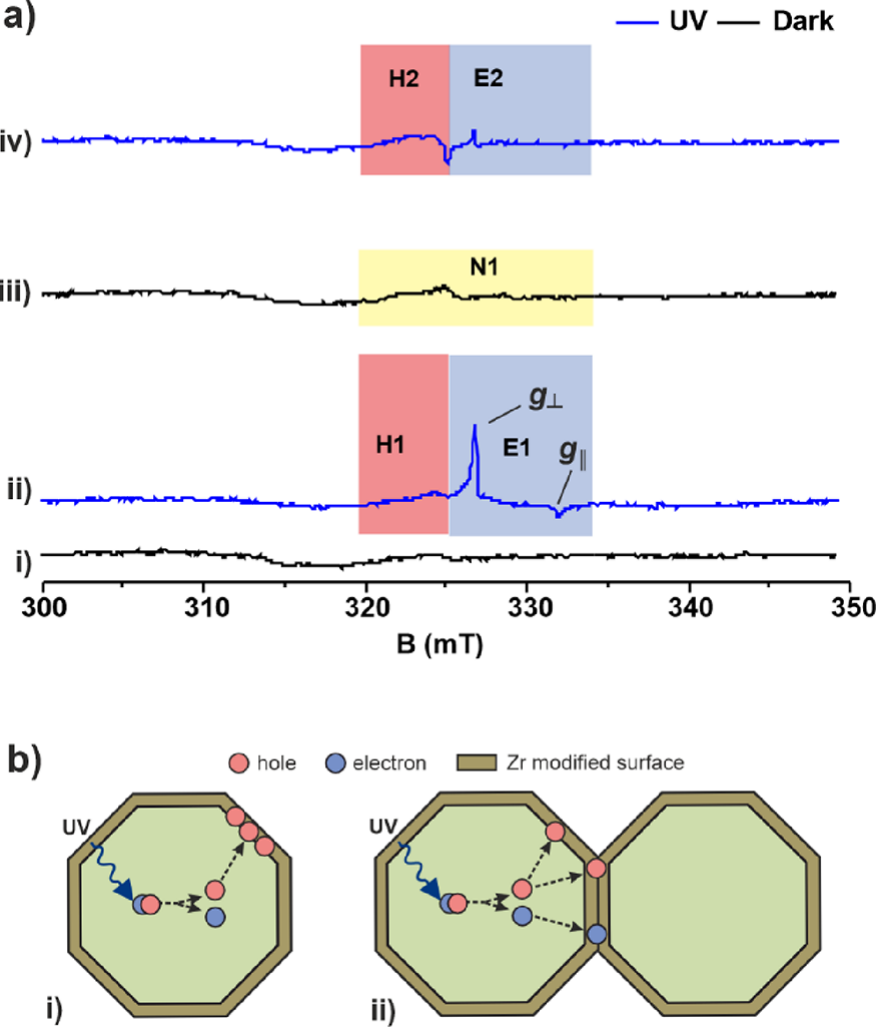
(a) EPR spectra of Zr-TiO_2_ (1.00 at%). (i) Before sintering in the dark; (ii) before sintering under continuous UV illumination; (iii) after sintering at 550 °C in the dark; (iv) after sintering at 550 °C under continuous UV illumination. All spectra were recorded at 77 K under vacuum (<10^–4^ mbar). (b) (i) Proposed processes on illumination before sintering. Surface adsorption of ZrF_6_^2–^ does not affect the intrinsic charge trapping processes in TiO_2_; (ii) proposed processes on illumination after sintering at 550 °C. Zr atoms at the interfacial region reduce charge trapping. All spectra were recorded at 77 K under high vacuum (<10^–4^ mbar).

The evolution of EPR signals attributable to trapping after Zr doping was used to conveniently study the effect of Zr precursor, concentration, and processing parameters. EPR of Zr-TiO_2_ (1.00 at%) before sintering, in the dark, and under UV illumination (Figure 7a(i) and (ii)) is essentially identical to TiO_2_ (Figure 6a(i) and (ii)), giving rise to similar sets of peaks, E1 (*g*_⊥_ = 1.991, *g*_∥_ = 1.960) and H1 (*g* ranging from 2.007 to 2.041), indicating that adsorbed H_2_ZrF_6_ does not affect the intrinsic trapping sites of TiO_2_. After sintering at 550 °C, the EPR spectrum of Zr-TiO_2_ (1.00 at%) in the dark (Figure 7a(iii)) is also very similar to TiO_2_ (Figure 6a(iii)), which also indicates that bulk F^–^ doping has not occurred that is known to give an intense signal in the dark characteristic of Ti^3+^.^62^ However, under illumination, there are pronounced differences between sintered Zr-TiO_2_ (Figure 7a(iv)) and sintered TiO_2_ (Figure 6a(iv)). The intensity of the signal (H2) attributed to trapped holes is significantly reduced for Zr-TiO_2_ with a smaller signal E revealed at a *g*-factor similar to but distinct from E1 (Figures 6a(ii) and 7a(ii)). The significant drop in the H2 trap state signals suggests effective modification of the interface and subsurface structure to remove hole trap states of TiO_2_, whereas bulk Ti^3+^ electron traps are unaffected (Figures 6b and 7b), consistent with theoretical prediction.

Of the three Zr concentrations investigated for Zr-TiO_2_ (0.12, 1.00, and 3.15 at%), all gave reduced EPR signal intensities of hole signals under illumination (Figure S14a–d) and Zr-TiO_2_ (1.00 at%), the greatest EPR signal reduction in the dark (Figure S14c). Three other Zr precursors, ZrCl_4_ 2THF, ZrO(NO_3_)_2_*x*H_2_O, and ZrOCl_2_*x*H_2_O, were also investigated (Figure S15–17), which showed significantly less reduction of the hole trapping signal of TiO_2_ compared to H_2_ZrF_6_. Of the Zr precursors investigated, H_2_ZrF_6_ is unique as a strong acid and therefore the effect of acid on TiO_2_ was studied by addition of nitric and acetic acids at equivalent pH to that used with H_2_ZrF_6_. In addition, Zr-TiO_2_ was also treated with ammonium bicarbonate to neutralize acidic moieties before sintering. On sintering at 550 °C, both acid-treated TiO_2_ and neutralized Zr-TiO_2_ showed some reduction in the H2 hole signal (Figures S18 and 19). These experiments suggest that acid can also induce structural changes to reduce hole trapping sites in addition to Zr doping. A further control examined addition of H_2_ZrF_6_ to sintered TiO_2_ followed by further sintering at 550 °C. No significant change in the EPR spectrum is observed (Figure S20) with retention of a large hole trapping signal (H2). This observation supports the hypothesis that the photogenerated hole trapping signals (H2) of sintered TiO_2_ are not located at the exposed surface but at the particle-particle interfaces.

Analogous surface treatment of commercial anatase TiO_2_ powder with 1 mM ethanolic H_2_ZrF_6_ prior to sintering was also studied. Sintering commercial anatase at 550 °C gives a similar EPR spectrum to sintered TiO_2_ with a distinct hole trapping region (Figure S21), although the EPR signals are more complex reflecting greater heterogeneity. Nevertheless, analogous treatment with 1 mM ethanolic H_2_ZrF_6_ prior to sintering also shows quenching of the hole trapping signal after sintering (Figure S22), suggesting that H_2_ZrF_6_ treatment can be used more generally to suppress localization of photogenerated holes at the grain boundaries of TiO_2_.

### Interparticle Charge Trapping of TiO_2_ and Zr-TiO_2_ Nanoporous Films

**Figure 8:**
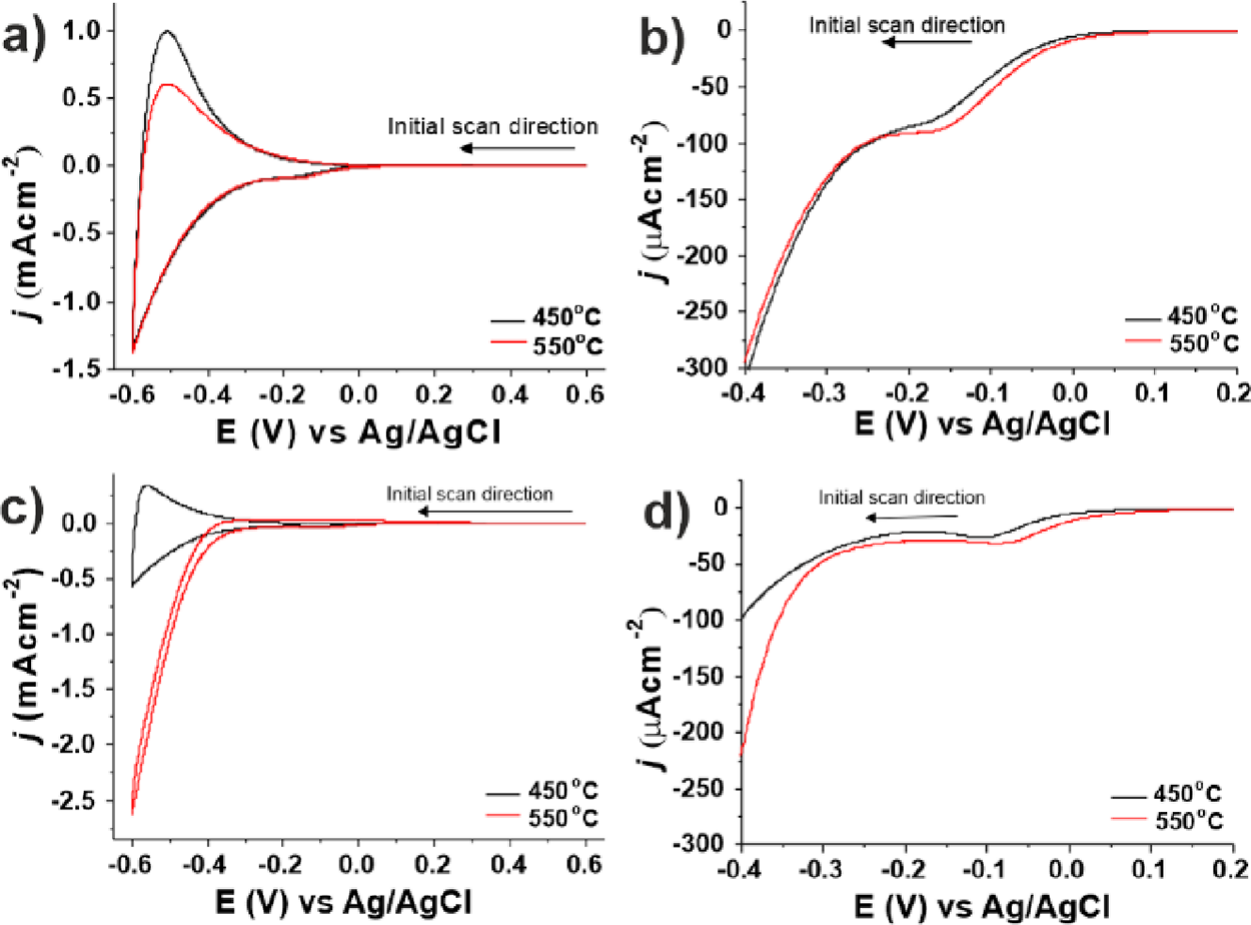
Cyclic voltammograms (first cycle) of (a, b) TiO_2_ films sintered at 450 and 550 °C and (c, d) Zr-TiO_2_ (1.00 at%) films sintered at 450 and 550 °C. (b) and (d) are expanded regions of the voltammograms from (a) and (b), respectively. Pt-mesh counter electrode, Ag/AgCl (3 M NaCl); reference electrode, electrolyte 100 mM HClO_4_ (aq); scan rate 20 mVs^–1^.

The electronic properties of nanoporous films were studied using electrochemical methods that support charge injection to investigate if reducing interfacial trapping of electrons improves electron mobility.^63−66^ Nanoporous films of TiO_2_ and Zr-TiO_2_ were prepared on conducting fluorine-doped tin oxide (FTO)-coated glass substrates using a doctor blading method (Figure S23). No organic binder was used in order to reduce contamination of the TiO_2_ surface and interfaces formed on sintering. Films were initially studied using cyclic voltammetry (CV) and EIS after first sintering in air at 450 °C and then subsequently at 550 °C to probe effects due to the film morphology and microstructure. CV of TiO_2_ films in 0.1 M HClO_4_ shows a cathodic current rapidly increasing after 0.1 V (vs Ag/AgCl) (Figure 8a), suggesting incremental electron filling of states below, and in, the conduction band (CB), as described in literature reports.^67−70^ For both sintering temperatures, a broad peak at −0.15 V is observed in the first cathodic scan (Figure 8b), which is absent in subsequent scans and reappears after additional sintering (Figure S24). The FWHM of this cathodic peak is more than 90 mV, which is consistent with that reported for filling of states below the CB of TiO_2_.^67,68^ Cathodic reduction of Ti^4+^ to Ti^3+^ is supported by the complementary intercalation of H^+^, which is limited by the film microstructure.^69^ Films sintered at 550 °C exhibit a shift to more positive potential by 30 mV (Figure 8b), the cause of which is currently unknown but presumably reflects changes to surface and interfacial states observed similarly for EPR data under illumination (Figure 5a). Similar peaks and behavior are observed for films of Zr-TiO_2_ (1.00 at%) (Figure 8c,d) sintered at 450 or 550 °C including a positive shift after sintering at 550 °C and peak loss after the first cycle (Figure S25).

The charge consumed in the cathodic half cycle is associated with the accumulation of electrons in TiO_2_ and reflects the trap state concentration at the surface and grain boundary region.^69^ Integration of the peaks and accounting for film thickness, density, and particle size allows the estimation of the average number of states filled per particle (Figure S26, Table S4). The data indicate that addition of Zr results in fewer states that can be filled. For example, TiO_2_ and Zr-TiO_2_ (1.00 at%) sintered at 550 °C are estimated to have 54 and 37 electrons per particle, respectively.

EIS of TiO_2_ and Zr-TiO_2_ (1.00 at%) nanoporous films sintered at 450 and 550 °C was performed in 0.1 M HClO_4_ solution at potentials across the main features of the voltammograms (Figure S27). The flat band potentials of TiO_2_ and Zr-TiO_2_ (1.00 at%) are −0.108 and – 0.179 V (vs Ag/AgCl), respectively (Figure S28), and at more positive potentials, the films behave as an insulator.^63^ At more negative potentials than ca. −0.3 V, a metallic-like behavior is observed as the CB is filled (Figure 8). Typical Nyquist plots of TiO_2_ and Zr-TiO_2_ (1.00 at%) (Figure S27) can be modeled using a transmission line model equivalent circuit. The main feature is a high-frequency elbow region that can be used to calculate the electronic conductivity of the film (ESI). The electronic transport within these nanoporous films is likely to be mainly diffusive in nature; hence, electron mobility can be extracted from the conductivity.

Comparison of the calculated conductivity and mobility of TiO_2_ and Zr-TiO_2_ (1.00 at%) from EIS measurements (Table 1) show similar values. Analogous measurements of Zr-TiO_2_ (3.15 at%) did exhibit a 40% increase in conductivity and mobility; however, it should be noted that the absolute magnitudes of all the values are at the lower end of the reported range measured for TiO_2_ morphologies using a range of techniques (Table S6). Nevertheless, the trends indicated in Table 1 suggest further optimization of Zr modification may be possible for increasing electron conductivity.

**Table 1:** Conductivity and the Mobility Values Obtained from EIS Measurements

sample	sintering temp. (°C)	conductivity (S cm^–1^) (× 10^–6^)	mobility (cm^2^ V^–1^ s^–1^) (× 10^–7^)
TiO_2_	450	1.41	0.88
TiO_2_	550	1.76	1.09
Zr-TiO_2_ (1.00 at% Zr)	450	1.55	0.97
Zr-TiO_2_ (1.00 at% Zr)	550	1.80	1.12
Zr-TiO_2_ (3.15 at% Zr)	450	2.19	1.37
Zr-TiO_2_ (3.15 at% Zr)	550	2.45	1.53

## Discussion

First principles modeling of select anatase surfaces and interfaces shows that spontaneous structural distortions occur, which result in electron and hole trap states within the band gap that can be alleviated by Zr doping. Of course, real crystals contain a very complex arrangement of surfaces and microstructures, which include dislocations, steps, edges, and vertices that cannot be exhaustively modeled individually, nor their combinations at interfaces or the presence of impurities present in real-world materials. Nonetheless, the general principle can be hypothesized that a sub-monolayer of atomically dispersed Zr can reduce the overall concentration of interfacial trap states (both electrons and holes), although this does not exclude effects of Zr on other types of defects not considered here. Experimentally, we have used faceted nanoparticles of anatase to increase the surface area available for study and have developed a method to disperse Zr at a sub-monolayer concentration. Compositional and structural analyses show that on sintering Zr is primarily located at particle surfaces and by implication the interfaces of TiO_2_ nanoparticles. EPR in the dark under equilibrium conditions indicates the absence of trapped electrons; however, under illumination significant hole-trapping occurs, which dominates. EPR of Zr-TiO_2_ in the presence of hole and electron scavengers and the addition of Zr to previously sintered TiO_2_ indicate that the photogenerated hole trap states are present in the region of the interparticle interfaces. These observations have implications for photocatalytic and photoelectrochemical applications where coatings of preformed powders and nanostructured electrode films are used to improve the efficiency often attributed to the reduction of surface defects and consequential reduction in electron–hole recombination.^71−73^ However, coatings also alter the surface chemistry and catalytic sites where the redox chemistry of the reaction of interest takes place, providing an alternative interpretation in the absence of further evidence.^74−76^ This study shows that, for the anatase nanoparticles investigated here, interfacial hole defects dominate on photoexcitation and doping sintered powders with Zr has little effect on these defects. Currently, the photocatalytic and photoelectrochemical relevance of these interfacial defects is not clear; however, doping before sintering and the formation of interfaces significantly reduce the interfacial defects, providing a simple methodology for potential further performance gains.

Nanoporous films were also prepared using simple doctor-blading followed by sintering, and electron injection using electrochemical methods was used to study the effect of Zr doping on trap states. CV measurements indicate the presence of states below the CB that can potentially trap electrons, and these are also reduced by Zr doping, which is reflected in the increased conductivity and mobility of the films as Zr doping is increased. However, the electrical conductivity and mobility of metal oxide films relevant to use in a device are determined by a complex array of interdependent phenomena including film morphology and thickness, particle size, interfacial contacts, and measurement area, which are represented by the range of reported values for TiO_2_ films for conductivity (10^–3^ to 10^–8^ Scm^–1^) and mobility (10^–7^ to 10 cm^2^ V^–1^ s^–1^) (Table S5). These are also very dependent on the timescale of the experimental method used to measure conductivity or mobility. The electrochemical technique used for conductivity and charge carrier mobility in the present study is based on a large perturbation at longer time scales reflective of their practical use in many devices. However, the nanoparticles used here give rise to porous films on FTO with a large number of particle interfaces giving inherently low conductivity and mobility. The mobility values extracted from these measurements are representative of all the underlying processes including trapping–detrapping, recombination, and intraparticle transport and do not necessarily reflect only the interparticle transport processes across the particle–particle interface. Nevertheless, the addition of Zr in a sub-monolayer concentration results in improvement in conductivity and mobility with further opportunities for optimization.

## Conclusions

First principles modeling has identified interfacial defects of anatase TiO_2_ arising from spontaneous structural distortions, which can be corrected in silico by doping with Zr. This hypothesis can be partially generalized experimentally to more complex surfaces and interfaces present in anatase nanocrystals sintered as powders and nanoporous films. Spectroscopic and electrochemical data indicate that Zr doping reduces the concentration of defects responsible for hole trapping on photoactivation and electron trapping on charge injection. For the latter, although bulk conductivity and electron mobility are improved, other factors detrimental to charge-carrier transport dominate in these films. Nevertheless, the present study describes simple methodology to achieve sub-monolayer coverage of TiO_2_ surfaces and interparticle interfaces and there is the opportunity for additional experimental optimization varying concentrations, solvents, and precursors to further reduce the concentration of defects. More generally, the theory-led strategy described here has been shown to successfully guide improvement in materials property, which in principle can be used to address defect correction in other nanoporous oxides, ultimately leading to improved efficiencies in systems incorporating conductive metal oxide films including solar and fuel cells.

## Supporting Information Available

Materials sources, characterization methods, estimation of Zr surface concentration, elemental analysis, adsorption isotherms, ATR-IR, EPR, XP, and UP spectroscopy, and electrochemistry (PDF)
