# Supplementary material for: Predictive Removal of Interfacial Defect-Induced Trap States between Titanium Dioxide Nanoparticles via Sub-Monolayer Zirconium Coating
Source: J Phys Chem C Nanomater Interfaces. 2022 Dec 23;127(1):660–71. doi: 10.1021/acs.jpcc.2c06927 (PMC9841567; doi:10.1021/acs.jpcc.2c06927)
Supplement: Supplementary file 1 — jp2c06927_si_001.pdf [file jp2c06927_si_001.pdf]

# Supporting Information

# Predictive Removal of Interfacial Defect-Induced Trap States between Titanium Dioxide Nanoparticles via Sub-Monolayer Zirconium Coating

Joyashish Debgupta,[†] Leonardo Lari,[‡] Mark Isaacs,[§,‖] John Carey,[‡] Keith P. McKenna,*[‡] Vlado K. Lazarov,[‡] Victor Chechik,*[†] Richard E. Douthwaite*[†]

[†]Department of Chemistry, University of York, York, YO10 5DD, UK.

[‡]Department of Physics, University of York, Heslington, York, YO10 5DD, UK

[§]HarwellXPS, R92 Research Complex at Harwell, Rutherford Appleton Laboratories, Harwell, Didcot, OX11 0QS, UK.

[‖]Department of Chemistry, University College London, 20 Gordon Street, London, WC1H 0AJ, UK.

**General**

**Materials:** Titanium tetrabutoxide (99+%), Nitrosyl tetrafluoborate (97%), hexane (all Alfa Aesar), oleic acid (90%), oleylamine (70%), hexafluorozirconic acid ($H_2ZrF_6$, 50 wt% in water) (all Sigma Aldrich), ethanol (99%), dimethyl formamide (all Fisher Scientific); $ZrOCl_2$. $xH_2O$ (Aldrich, 99.99%); $ZrO(NO_3)_2$. $xH_2O$ (Aldrich, 99%); $ZrCl_4$. 2THF (Alfa Aeaser); Acetic acid (HOAc), Nitric acid ($HNO_3$); were all used as received without further purification.

**Estimation of zirconium surface concentration of Zr-$TiO_2$**

Assuming a cubic nanoparticle (NP) of edge length = L

Volume of NP: $NP_V = L^3$

Surface area of NP: $NP_A = 6L^2$

Unit cell of anatase[1] = 136.24 Å$^3$ with Z = 4; Volume of single '$TiO_2$' unit of anatase $TiO_{2V}$ = 34.06 Å$^3$

Number of Ti atoms in a NP: $\mathrm{NP_{T_i}} = \frac{\mathrm{NP}_v}{\mathrm{TiO2v}}$

Number of Zr atoms in a NP: $NP_{Zr}$

Atomic fraction of Zr: $f_{Zr} = \frac{\mathrm{NP_{Zr}}}{\mathrm{NP_{T_i}}}$

Number of Zr ions added per NP: $NP_{Zr} = f_{Zr}\, NP_{Ti}$

Maximum surface concentration of Zr: $\mathrm{SC}_{Zr} = \frac{\mathrm{NP_{Zr}}}{\mathrm{NP}_A} = \frac{f_{\mathrm{Zr}}.\mathrm{NP_{Ti}}}{6\mathrm{L}^2} = \frac{f_{\mathrm{Zr}}.\mathrm{NP_v}}{6\mathrm{L}^2.\mathrm{TiO2_v}} = \frac{f_{\mathrm{Zr}}.\mathrm{L}^3}{6\mathrm{L}^2.\mathrm{TiO2_v}} = \frac{f_{\mathrm{Zr}}.\mathrm{L}}{6.\mathrm{TiO2_v}}$

For L = 15 nm (from SEM) and $f_{Zr}$ = 0.01 (From ICP-MS), $SC_{Zr}$ = 0.007 Å$^{-2}$ = 0.7 nm$^{-2}$

The 101 anatase surface contains a Ti surface concentration, $SC_{Ti}$ = 5-10 nm$^{-2}$

Therefore the concentration of Zr is sub-monolayer and insufficient to coat a $TiO_2$ nanoparticle.

**Elemental analysis**

**Table S1.** CHN microanalysis of (a) **$SL-TiO_2$** and (b) **$TiO_2$**.

**$SL-TiO_2$**

| Element | % C | % H | % N | % remaining |
|---|---|---|---|---|
| Expt 1 | 8.89 | 1.59 | 0.29 | 89.22 |
| Expt 2 | 8.86 | 1.45 | 0.15 | 89.54 |

**$TiO_2$**

| Element | % C | % H | % N | % remaining |
|---|---|---|---|---|
| Expt 1 | 2.33 | 0.40 | 0 | 97.27 |
| Expt 2 | 1.43 | 0.24 | 0 | 98.33 |

**Table S2.** Ti and Zr ICP-MS analysis of **$Zr-TiO_2$**.

| | % Ti | % Zr |
|---|---|---|
| **$Zr-TiO_2$ (20 μM)**[a] | 55.56 | 0.12 |
| **$Zr-TiO_2$ (1 mM)** | 54.78 | 1.00 |
| **$Zr-TiO_2$ (5 mM)** | 55.22 | 3.15 |

[a] Concentration of Zr solution used during synthesis (see above).

**Table S3.** Ti and Zr XPS compositional analysis of **$Zr-TiO_2$** before and after sintering.

| | % Ti[a] | % Ti[b] | % Zr[a] | % Zr[b] |
|---|---|---|---|---|
| **$Zr-TiO_2$ (20 μM)**[a] | 23.10 | 23.10 | 0.05 | 0 |
| **$Zr-TiO_2$ (1 mM)** | 23.47 | 23.85 | 0.32 | 0.22 |
| **$Zr-TiO_2$ (5 mM)** | 21.94 | 23.07 | 0.92 | 0.84 |

[a]before sintering, [b]after sintering at 550 °C.

**ATR-IR spectroscopy**

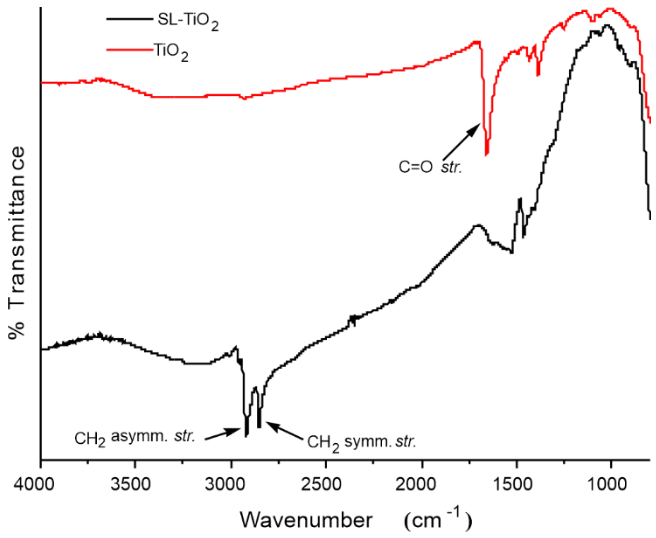


**Figure S1.** ATR-IR spectra of **SL-TiO$_2$** and **TiO$_2$** after drying under vacuum at room temperature. Disappearance of -$CH_2$ *str.* peak of **SL-TiO$_2$** shows removal of oleic acid and oleylamine surface ligands. For **TiO$_2$** the absence of peaks at 1084 cm$^{-1}$ (for $BF_4^-$) or in the range 2100-2200 cm$^{-1}$ (for $NO^+$) suggests no residual $NOBF_4$. For **TiO$_2$** the sharp peak at 1654 cm$^{-1}$ is attributable to adsorbed DMF used for washing $NOBF_4$ treated nanoparticles. Spectra are offset for clarity.

**TEM**

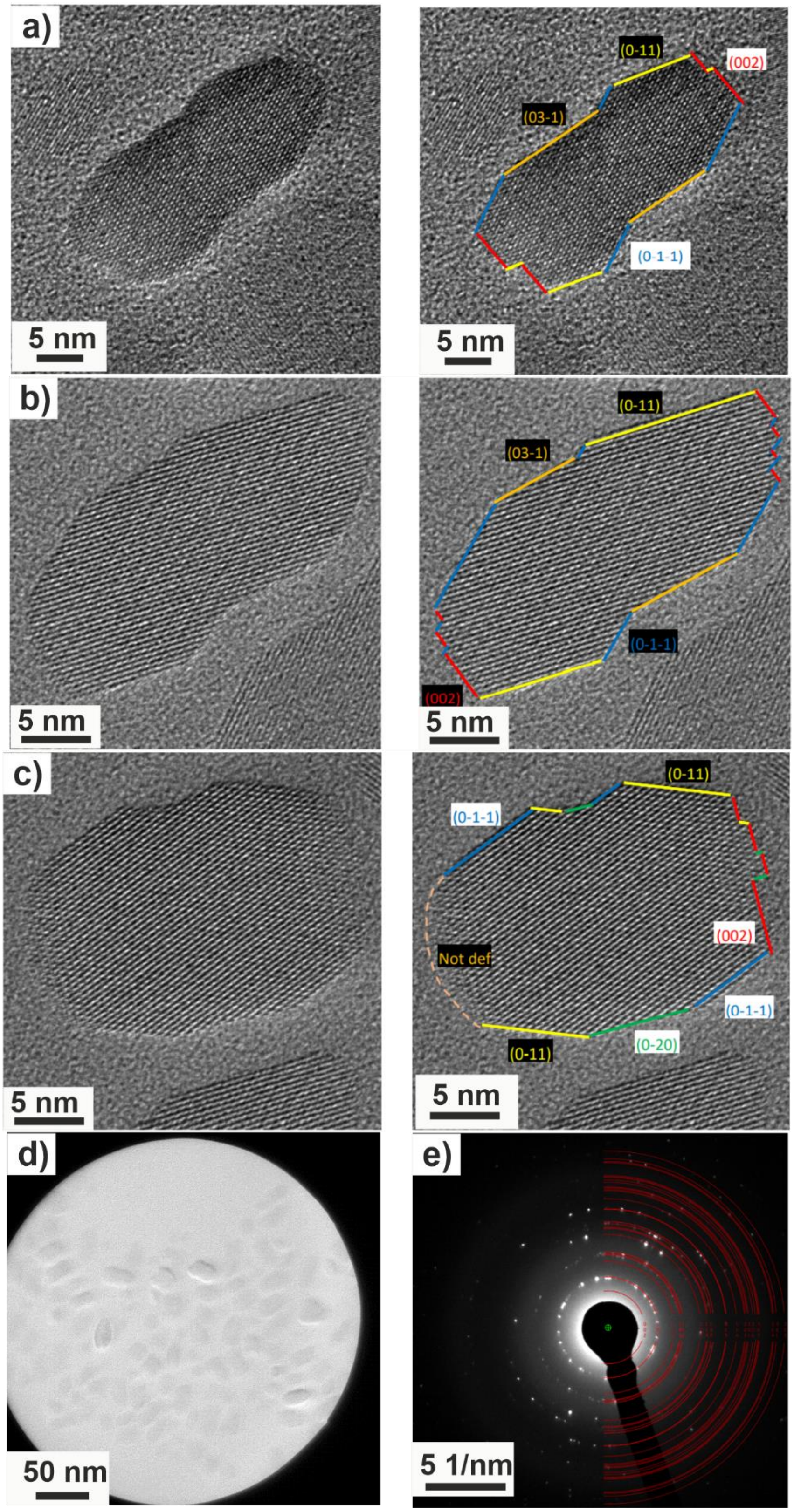


**Figure S2.** a-c) TEM determination of facets for **SL-TiO$_2$** nanoparticles. a-c are three representative nanoparticles from the same batch). In the space group I4$_1$/amd facets (0,-1,1) and (0,-1,-1) are equivalent to the {1,0,1} family of planes; d) Selected area for diffraction pattern shown in (e); (e) experimental SAED pattern with calculated diffraction rings for anatase.

**Powder X-ray diffraction analysis**

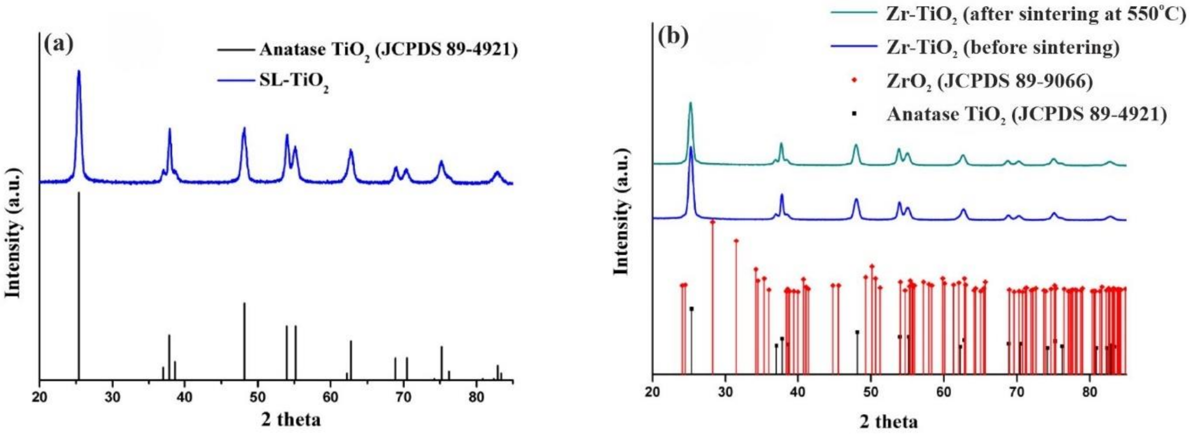


**Figure S3.** Powder X-ray diffraction diffractogram of (a) **SL-TiO$_2$** and (b) **Zr-TiO$_2$** before and after sintering in air at 550˚C.

**N$_2$ adsorption isotherms (BET analysis)**

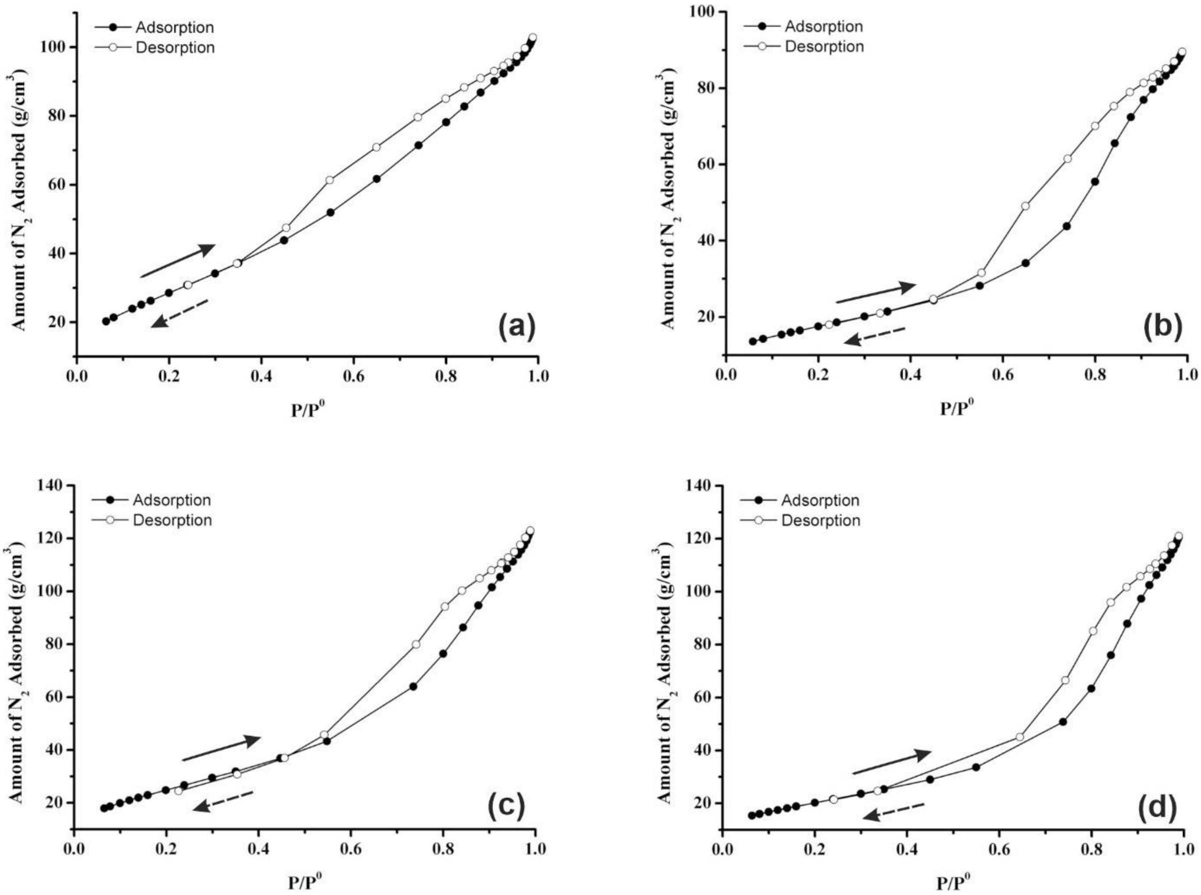


**Figure S4.** N$_2$ adsorption isotherms of (a-b) **TiO$_2$** and (c-d) **Zr-TiO$_2$** (1.00 at%); (a, c) before and (b, d) after sintering at 550 $^oC$ in air, respectively; solid and broken arrows represent the direction of adsorption and desorption respectively.

**EDX analysis of Zr-$TiO_2$**

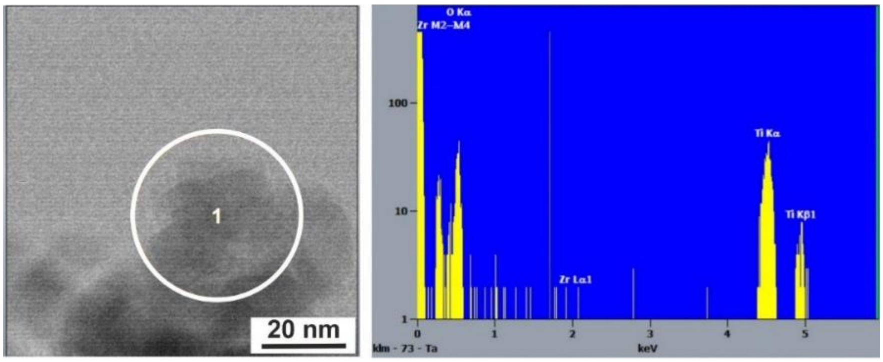


**Figure S5.** (a) EDX-SEM elemental mapping for **Zr-$TiO_2$** (before sintering) and (b) STEM elemental analysis for **Zr-$TiO_2$** after sintering in air at 550˚C showing Zr at background level.

## X-ray and Ultraviolet Photoelectron Spectroscopy

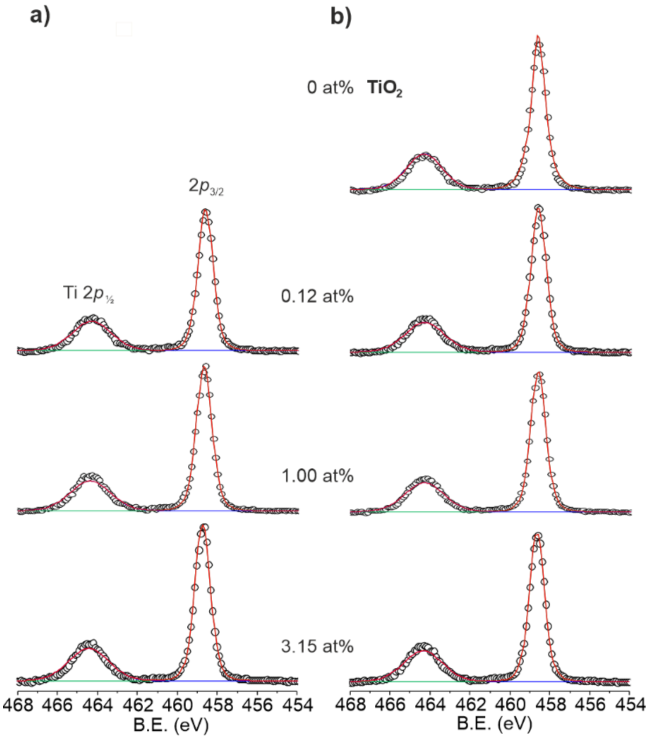


**Figure S6.** Ti 2*p* X-ray photoelectron spectra (a) unsintered and (b) sintered (550 °C) **$TiO_2$** and **Zr-$TiO_2$** with Zr-doping as indicated. Open circle (o) raw data, red line (—) overall fit, green (—) and blue (—) lines peak 1 and peak 2 fits respectively.

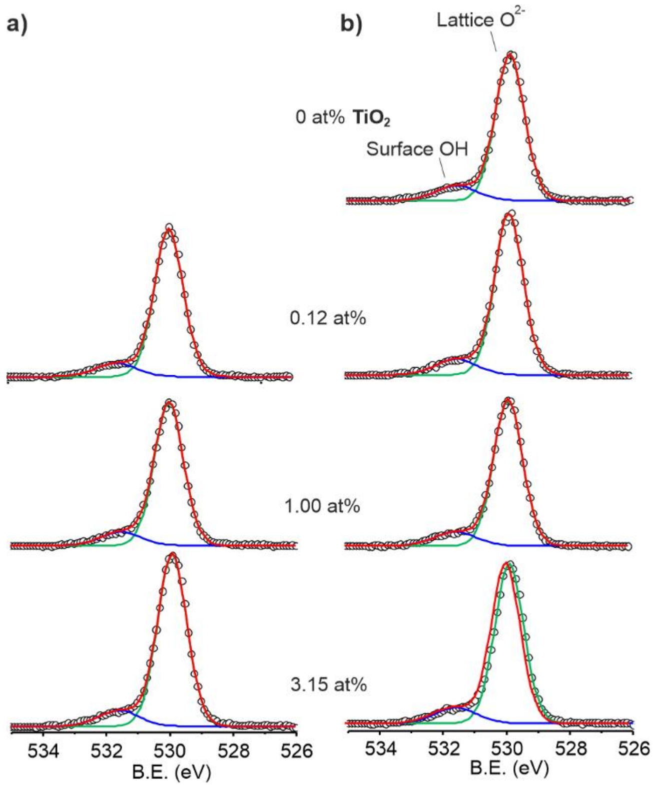


**Figure S7.** O 1*s* X-ray photoelectron spectra of (a) unsintered and (b) sintered (550 °C) **$TiO_2$** and **Zr-$TiO_2$** with Zr-doping as indicated. Open circle (o) raw data, red line (—) overall fit, green (—) and blue (—) lines peak 1 and peak 2 fits respectively.

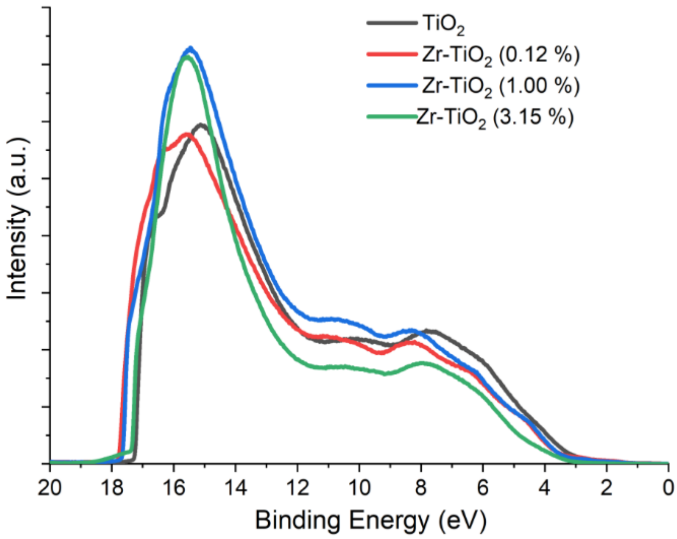


**Figure S8:** Ultraviolet photoelectron spectra (UPS) of **$TiO_2$** and **Zr-$TiO_2$** (after sintering at 550 °C).

**EPR Spectroscopy**

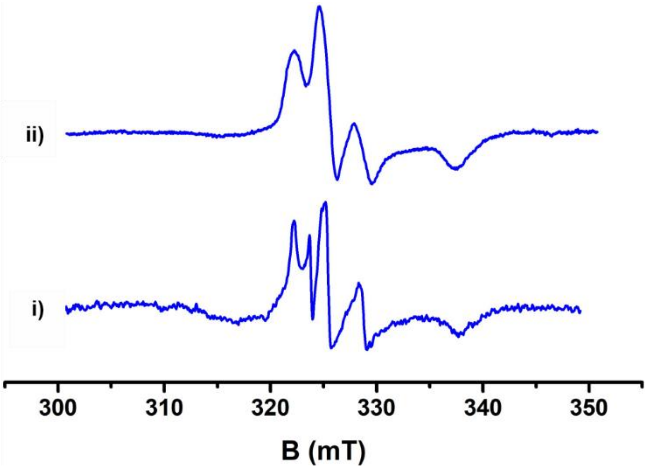


**Figure S9.** EPR spectra of (i) $\mathbf{TiO_2}$ sintered at 550˚C in air and (ii) **SL-**$\mathbf{TiO_2}$ sintered at 550˚C; all spectra were recorded in the dark at 77 K under vacuum (< $10^{-4}$ mbar).

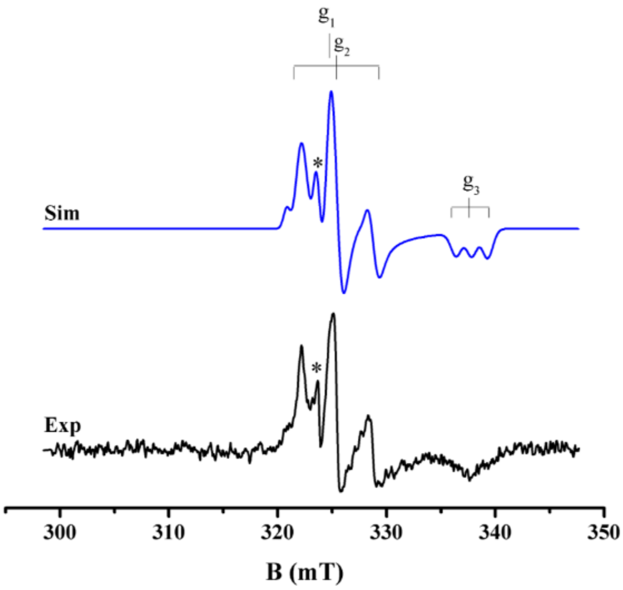


| Species | $g_1$ | $g_2$ | $g_3$ | $A_1$ (G) | $A_2$ (G) | $A_3$ (G) |
|---|---|---|---|---|---|---|
| NO | 2.0037 | 1.9997 | 1.92635 | 0.714 | 32.733 | 13.851 |
| $N_b$ | 2.01141 | 2.01076 | 2.00652 | 2.228 | 4.255 | 35.203 |

**Figure S10.** Experimental and simulated EPR spectra of $\mathbf{TiO_2}$, sintered at 550 °C, in the dark at 77 K under vacuum (< $10^{-4}$ mbar). (*) indicates a peak corresponding to bulk nitrogen ($N_b$ type) in N-doped $TiO_2$.[2]

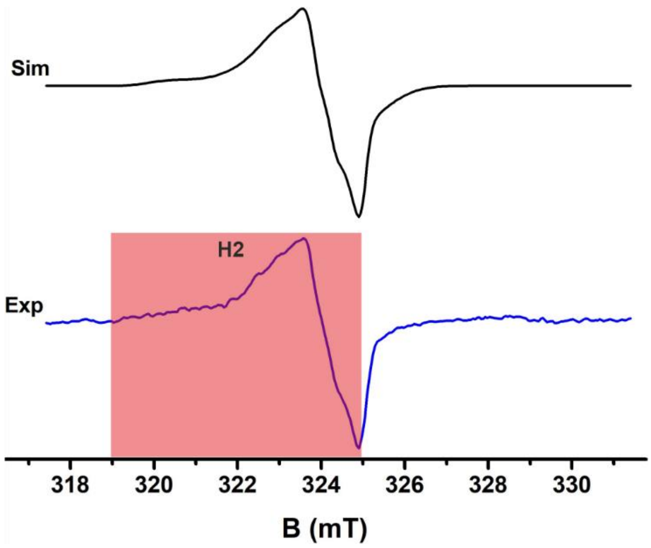


| Signal | Plausible species | System Weight (%) | $g_1$ | $g_2$ | $g_3$ | Sys. $g_1$ strain | Sys. $g_2$ strain | Sys. $g_2$ strain |
|---|---|---|---|---|---|---|---|---|
| H2 | $Ti^{4+} - O_2^-$ | 19 | 2.00286 | 2.00707 | 2.03185 | 0 | 0.00125 | 0.007392 |
| | $Ti^{4+} - O^{2-} - Ti^{4+} - O^-(O_S^-)$ | 37 | 2.00403 | 2.01005 | 2.02127 | 0 | 0 | 0.012506 |
| | $Ti^{4+} - O^- - Ti^{4+} - OH^-(O_B^-)$ | 44 | 2.002 | 2.01014 | 2.01308 | 0 | 0 | 0 |

$O_S$ = surface species, $O_B$ = subsurface lattice oxygen

**Figure S11.** Experimental and simulated EPR spectra of **$TiO_2$** after sintering in air at 550 °C under continuous UV illumination at 77 K under vacuum (< $10^{-4}$ mbar). Table showing the parameters obtained from fitting. Assignment of species based on reported g-values.[3]

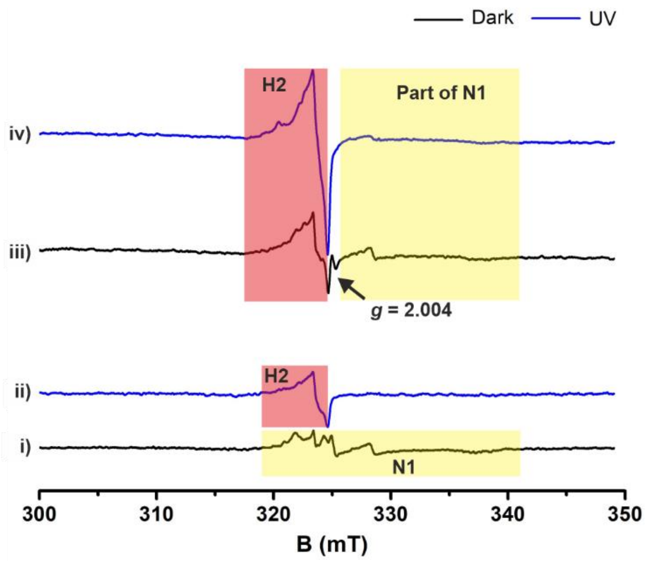


**Figure S12.** EPR of sintered **$TiO_2$** (i) in the dark before exposure to $O_2$; (ii) under continuous UV illumination before exposure to $O_2$; (iii) in the dark after exposure to 1 bar $O_2$ at 77K followed by pumping out excess $O_2$ at 77 K; (iv) under continuous UV illumination after exposure to 1 bar $O_2$ at 77K followed by pumping out excess unreacted $O_2$ at 77 K in the dark. Spectra (iii) shows a peak with *g* = 2.004 overlaying the N1 signal attributable to adsorbed peroxide species. All spectra were recorded at 77 K under high vacuum (< $10^{-4}$ mbar).

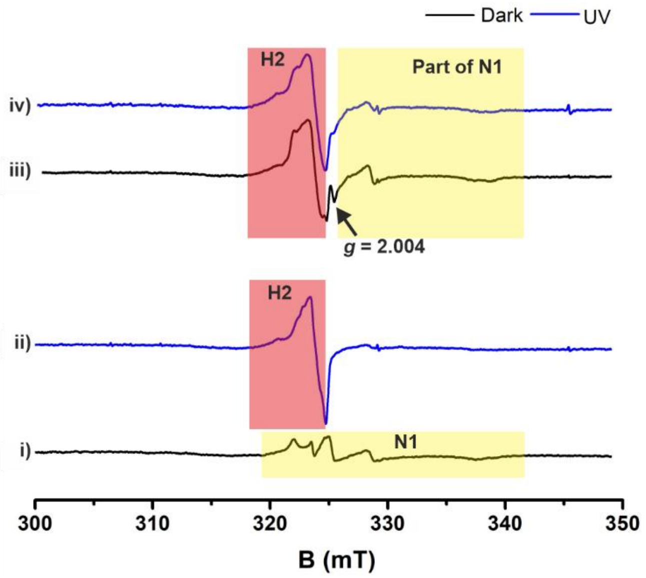


**Figure S13.** EPR of sintered **$TiO_2$** (i) in the dark before exposure to 2-propanol vapour; (ii) under continuous UV illumination before exposure to 2-propanol vapour; (iii) in the dark after exposure to 2 mbar 2-propanol vapour at 77K followed by pumping out excess 2-propanol vapour at 77 K; (iv) under continuous UV illumination after exposure to 2 mbar 2-propanol vapour at 77 K in the dark followed by pumping out excess unreacted 2 mbar 2-propanol vapour at 77 K in the dark. All spectra were recorded at 77 K under high vacuum (< $10^{-4}$ mbar).

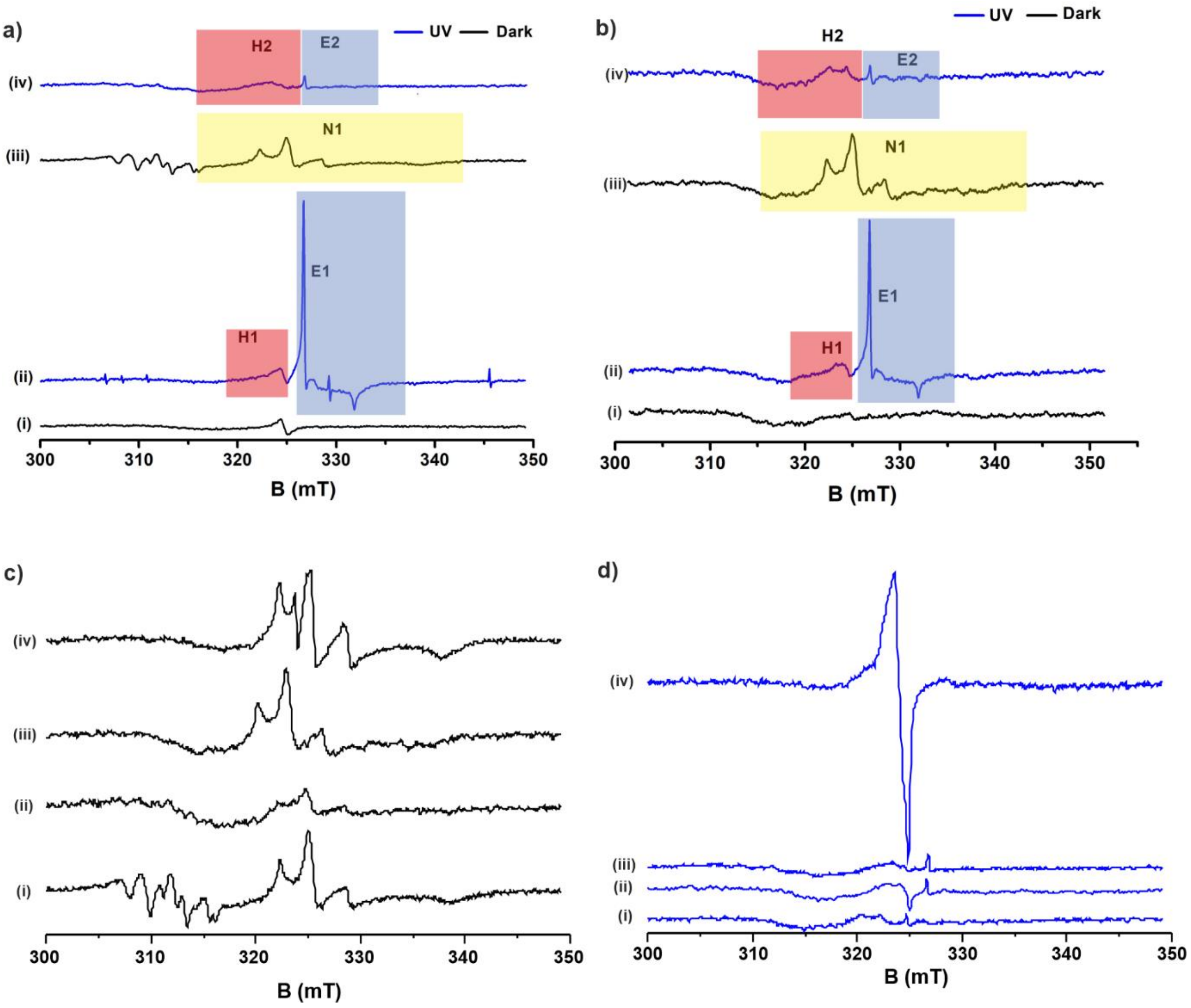


**Figure S14.** EPR spectra of (a) **Zr-TiO₂** (0.12 at%). (i) Before sintering in the dark; (ii) before sintering under continuous UV illumination; (iii) after sintering at 550˚C in the dark; (iv) after sintering at 550˚C under continuous UV illumination. (b) **Zr-TiO₂** (3.15 at%). (i) Before sintering in the dark; (ii) before sintering under continuous UV illumination; (iii) after sintering at 550˚C in the dark; (iv) after sintering at 550˚C under continuous UV illumination. (c) EPR in the dark of sintered (550 ºC) (i) **Zr-TiO₂** (0.12 at%); (ii) **Zr-TiO₂** (1.00 at%); (iii) **Zr-TiO₂** (3.15 at%); (iv) **TiO₂**. (d) EPR under continuous illumination of sintered (550 ºC) (i) **Zr-TiO₂** (0.12 at%); (ii) **Zr-TiO₂** (1.00 at%); (iii) **Zr-TiO₂** (3.15 at%); (iv) **TiO₂**. The presence of Zr at all concentrations suppresses the hole trapping signal (H2) of **TiO₂**. All spectra were recorded at 77 K under vacuum (< $10^{-4}$ mbar).

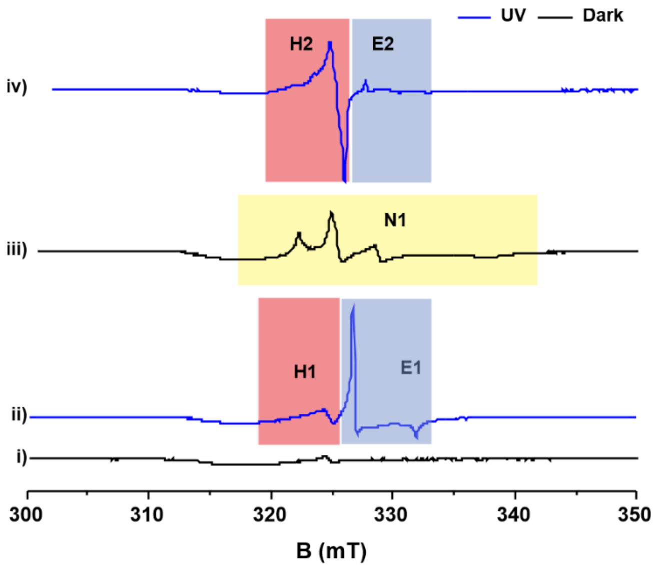


**Figure S15.** EPR spectra of **$TiO_2$** treated with 1 mM $ZrCl_4$.2THF. (i, ii) before sintering; (iii, iv) after sintering at 550˚C in air. All spectra were recorded at 77 K under vacuum (< $10^{-4}$ mbar).

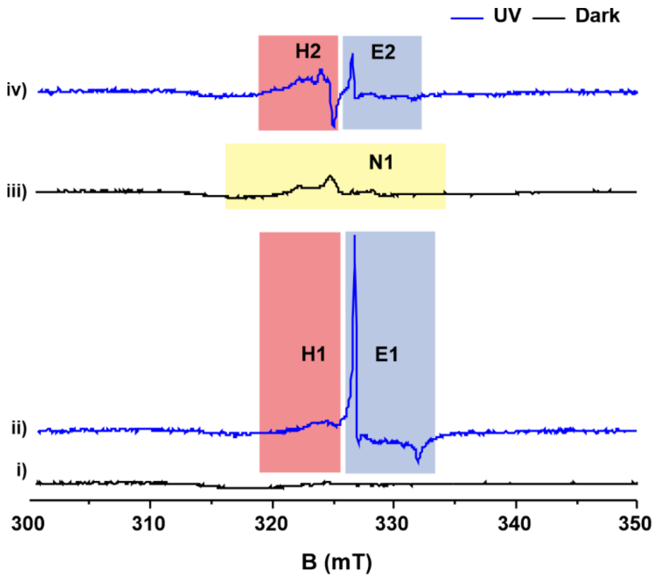


**Figure S16.** EPR spectra of **$TiO_2$** treated with 1 mM $ZrO(NO_3)_2.xH_2O$. (i, ii) before sintering; (iii, iv) after sintering at 550˚C in air. All spectra were recorded at 77 K under vacuum (< $10^{-4}$ mbar).

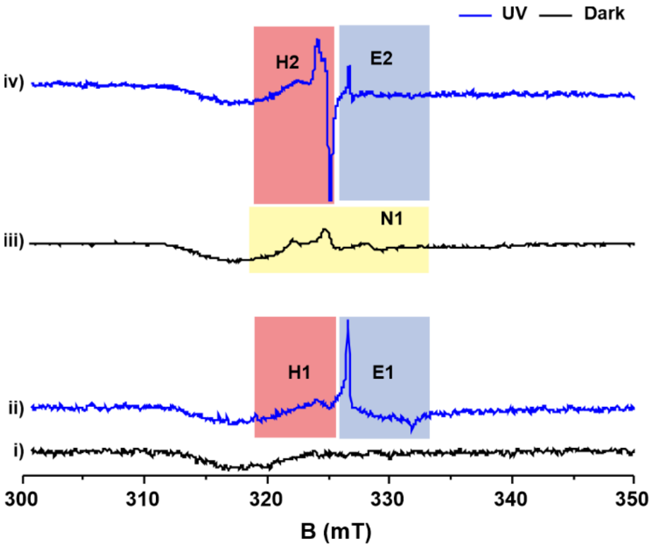


**Figure S17.** EPR spectra of **$TiO_2$** treated with 1 mM $ZrOCl_2.xH_2O$. (i, ii) before sintering; (iii, iv) after sintering at 550˚C in air. All spectra were recorded at 77 K under vacuum (< $10^{-4}$ mbar).

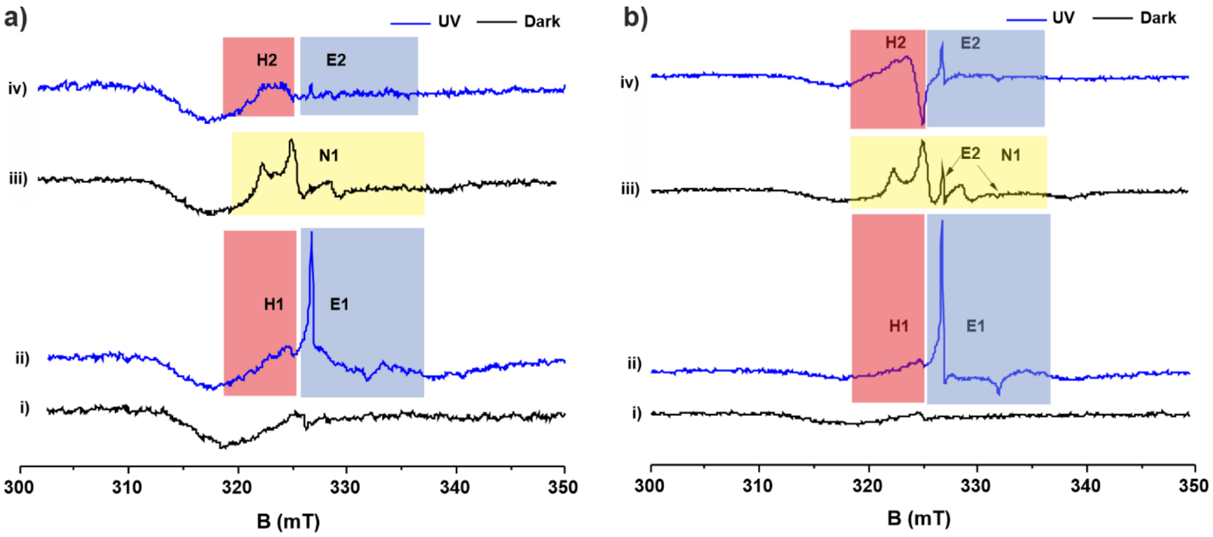


**Figure S18.** Effect of acid on the EPR spectra of **$TiO_2$**. (a) added 2 mM ethanolic $HNO_3$; (b) added 1 M ethanolic HOAc; (i,ii) before sintering; (iii, iv) after sintering at 550˚C in air for 1 h inside the EPR tube. All spectra were recorded at 77 K under vacuum (< $10^{-4}$ mbar).

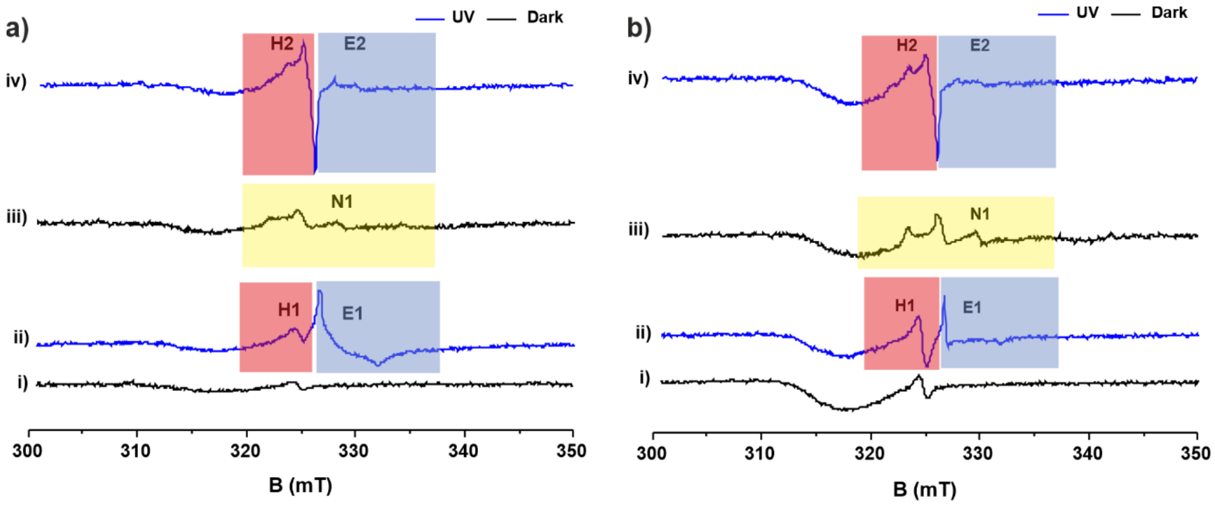


**Figure S19.** EPR spectra of 1 mM ethanolic $H_2ZrF_6$ treated **$TiO_2$** after washing with 1 mM $NH_4HCO_{3(aq)}$ to neutralize excess acid on the surface of **$TiO_2$**-$H_2ZrF_6$ (1 mM). (a) **$TiO_2$**-$H_2ZrF_6$ washed with only deionised water, (b) **$TiO_2$**-$H_2ZrF_6$ (1 mM) washed with 1 mM aq. $NH_4HCO_3$ solution; (i, ii) before sintering; (iii, iv) after sintering at 550˚C in air for 1 h inside the EPR tube. All spectra were recorded at 77 K under vacuum (< $10^{-4}$ mbar).

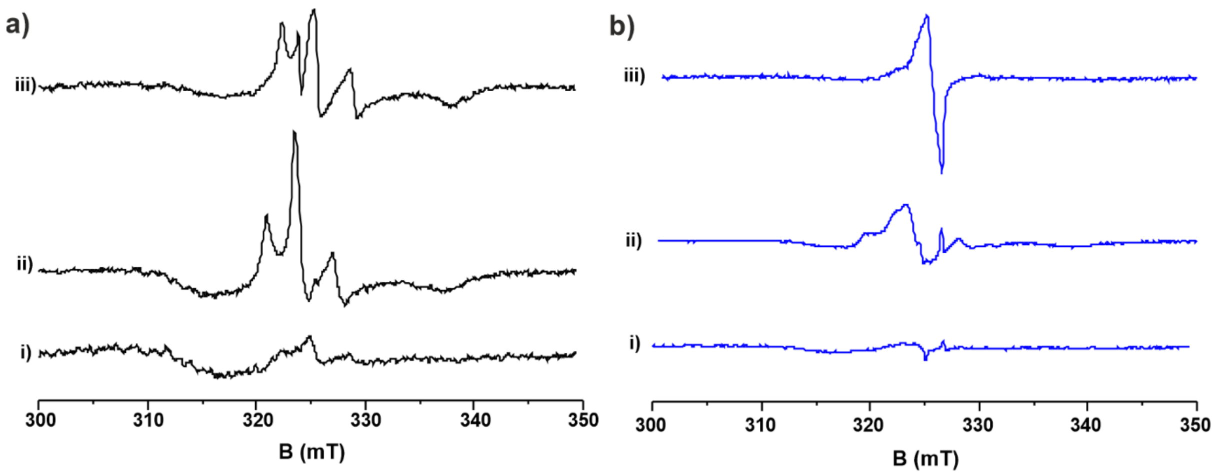


**Figure S20.** (a) Dark EPR spectra of; i) sintered (550 °C) **Zr-$TiO_2$** (1.00 at %); (ii) sintered **$TiO_2$** treated with 1 mM $H_2ZrF_6$ followed by sintering at 550˚C in air; (iii) sintered (550 °C) **$TiO_2$**. (b) EPR spectra under continuous UV illumination of; i) sintered (550 °C) **Zr-$TiO_2$** (1.00 at %); (ii) sintered **$TiO_2$** treated with 1 mM $H_2ZrF_6$ followed by sintering at 550˚C in air; (iii) sintered (550 °C) **$TiO_2$**. All spectra were recorded at 77 K under high vacuum (< $10^{-4}$ mbar).

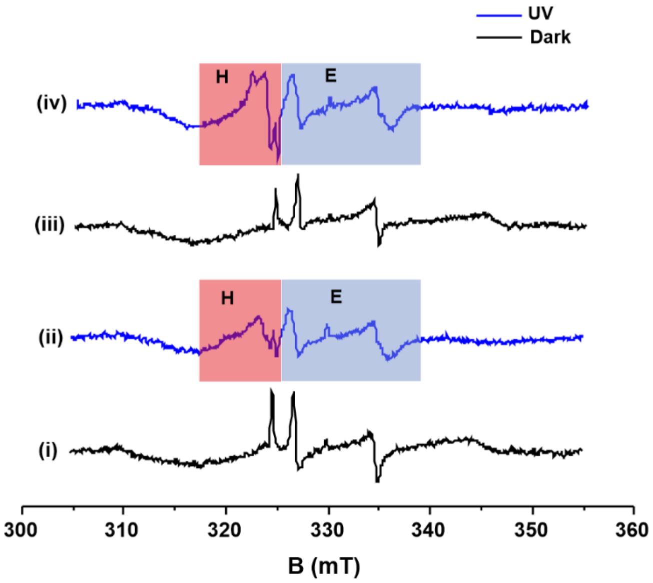


**Figure S21.** EPR spectra of commercial anatase $TiO_2$ nanoparticles. (i, ii) without sintering (iii, iv) after sintering at 550˚C in air. All spectra were recorded at 77 K under high vacuum (< $10^{-4}$ mbar).

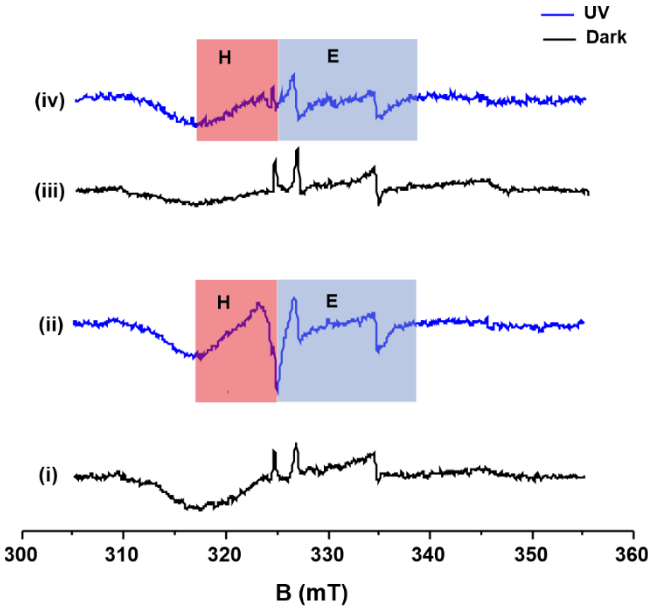


**Figure S22.** EPR spectra of commercial anatase $TiO_2$ nanopowder treated with 1 mM ethanolic $H_2ZrF_6$ solution. (i, ii) before sintering and (iii, iv) after sintering at 550˚C in air. All spectra were recorded at 77 K under vacuum (< $10^{-4}$ mbar).

## Electrochemistry

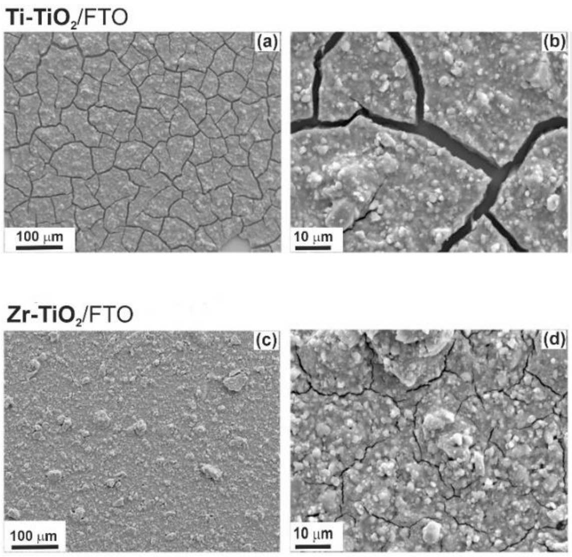


**Figure S23** a-b) plane view SEM images of **$TiO_2$** films and c-d) **Zr-$TiO_2$** (1.00 at%) films on FTO substrates after sintering at 550˚C in air.

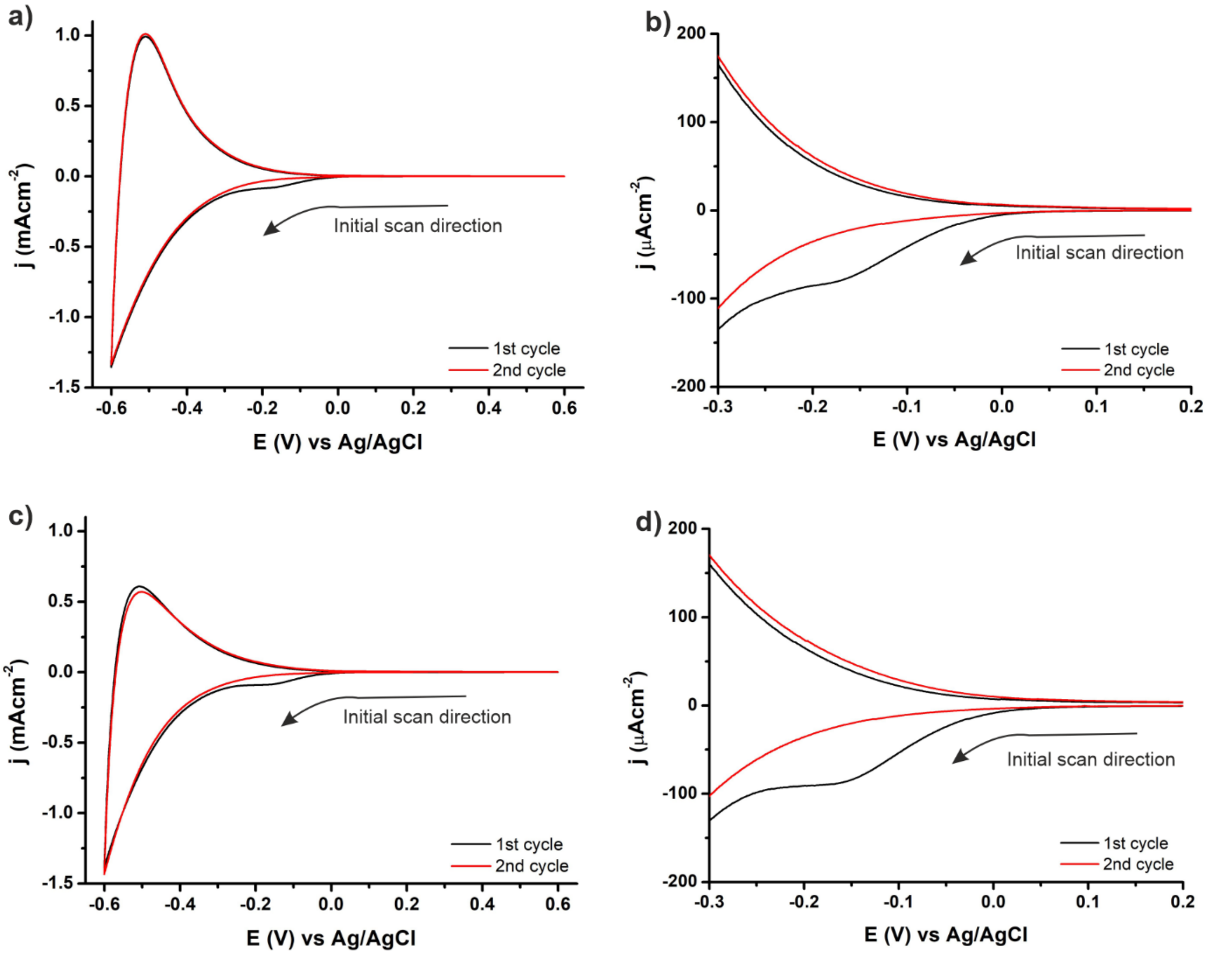


**Figure S24.** Effect of cycling **$TiO_2$**/FTO films sintered at (a) 450˚C and then subsequently at (b) 550˚C; (b) and (d) are expanded regions of (a) and (c) respectively. Pt-mesh counter electrode, Ag/AgCl (3 M NaCl) reference electrode, electrolyte of $N_2$ purged 100 mM $HClO_4$ (aq), scan rate-20 $mVs^{-1}$.

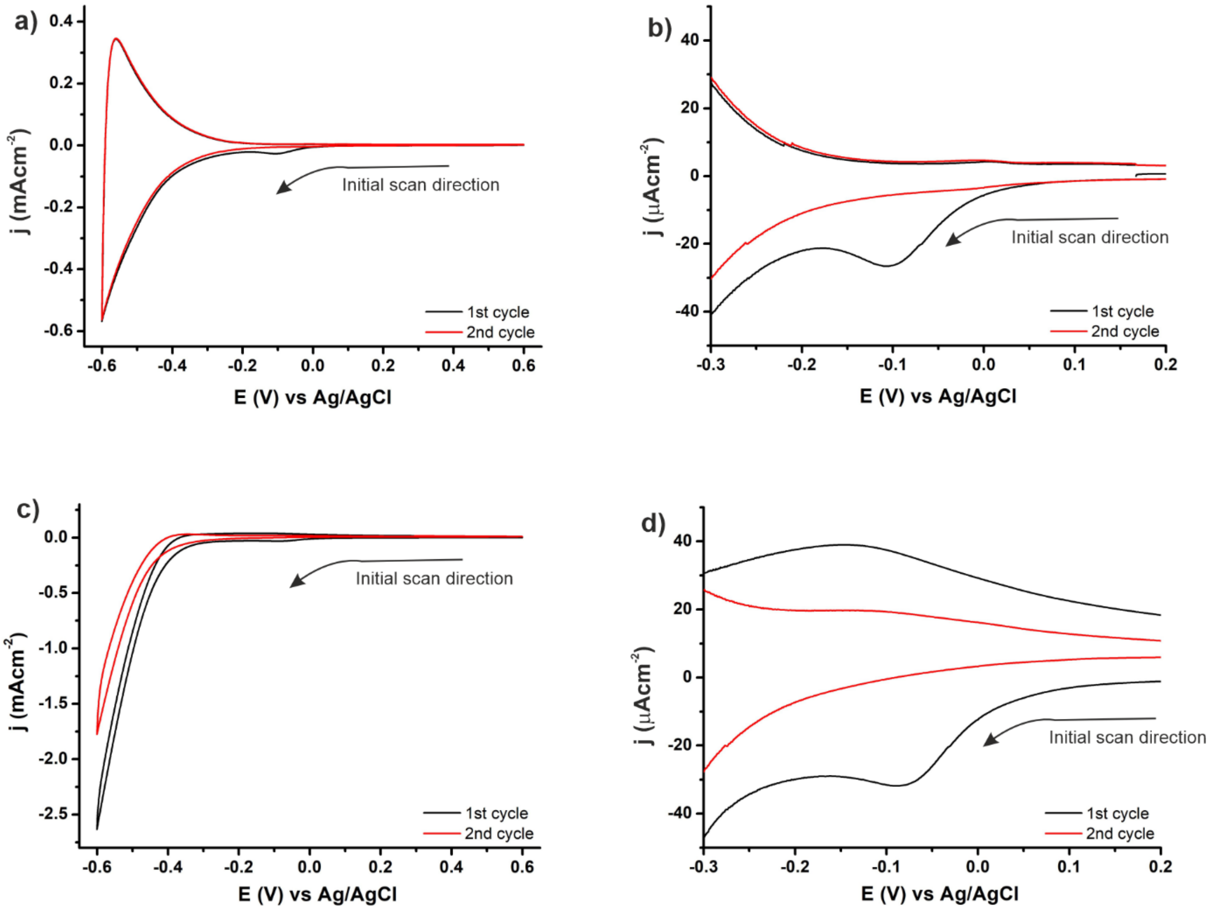


**Figure S25.** Effect of cycling **Zr-TiO$_2$** (1.00 at%))/FTO films sintered at (a) 450 and the same film at (c) 550 °C; (b) and (d) are expanded regions of (a) and (c) respectively. Pt-mesh counter electrode, Ag/AgCl (3 M NaCl) reference electrode, electrolyte of $N_2$ purged 100 mM $HClO_4$ (aq), scan rate-20 mVs$^{-1}$.

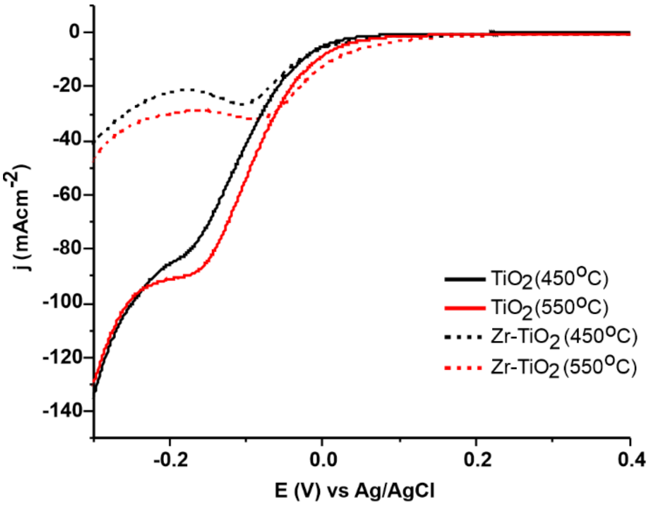


**Figure S26.** Comparison (superimposed data from Figs 9b) and 9d)) of cyclic voltammograms of **TiO$_2$** and **Zr-TiO$_2$** (1.00 at%)/FTO films sintered at 450 and 550 °C. Pt-mesh counter electrode, Ag/AgCl (3 M NaCl) reference electrode, electrolyte of $N_2$ purged 100 mM $HClO_4$ (aq).

**Table S4:** Number of electrons associated with the grain boundary peaks for individual particles as calculated from cyclic voltammetry experiment

| Film | Sintering Temp. (˚C) | Integrated cathodic charge (mC/cm$^2$) | | Charge for cathodic peak (μC/cm$^2$) | Mass of $TiO_2$ (mg) | A (mm$^2$) | t (μm) | P (%) | Electrons per particle[a] |
|---|---|---|---|---|---|---|---|---|---|
| | | 1$^{st}$ cycle | 2$^{nd}$ cycle | | | | | | |
| **$TiO_2$** | 450 | 9.44 | 8.66 | 559.7 | 1.75 | 65 | 9.8 ± 2.5 | 70.3 | 41 |
| **$TiO_2$** | 550 | 9.41 | 8.5 | 747.2 | 1.75 | 65 | 9.8 ± 2.5 | 70.3 | 54 |
| **Zr-$TiO_2$** | 450 | 3.54 | 3.15 | 181.0 | 0.60 | 50 | 6.3 ± 2.5 | 58.9 | 25 |
| **Zr-$TiO_2$** | 550 | 12.75 | 7.36 | 267.6 | 0.60 | 50 | 6.3 ± 2.5 | 58.9 | 37 |

[a] *Assuming cubic particles with average length of 20 nm*

*A= geometrical area, t=thickness of the film, P=porosity of the film*

## Electrochemical Impedance Spectroscopy (EIS)

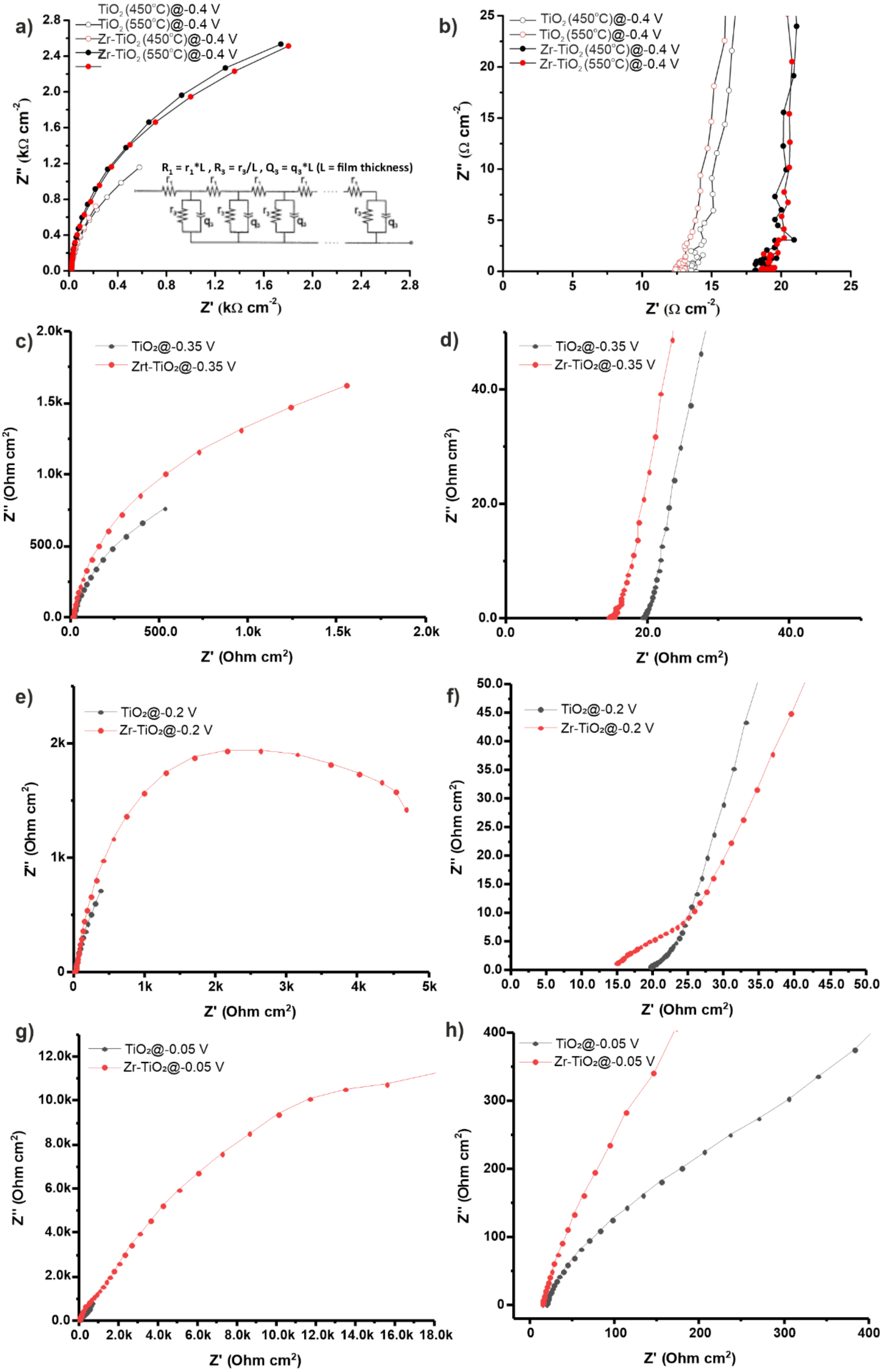


**Figure S27.** Nyquist plots for FTO supported nanoporous films of $\mathbf{TiO_2}$ and $\mathbf{Zr\text{-}TiO_2}$ (1.00 at%) at (a,b) -0.4 V, (c,d) -0.35 V; (e,f) -0.2 V; (g,h) -0.05 V (vs Ag/AgCl); a inset) transmission line model equivalent circuit R1 is the resistance for charge carrier transport within the film, R3 and Q3 are the charge

transfer resistance and corresponding capacitance, respectively across the $TiO_2$-electrolyte interface. Pt-mesh counter electrode, Ag/AgCl (3 M NaCl) reference electrode, electrolyte is air saturated 100 mM $HClO_4$ (aq).

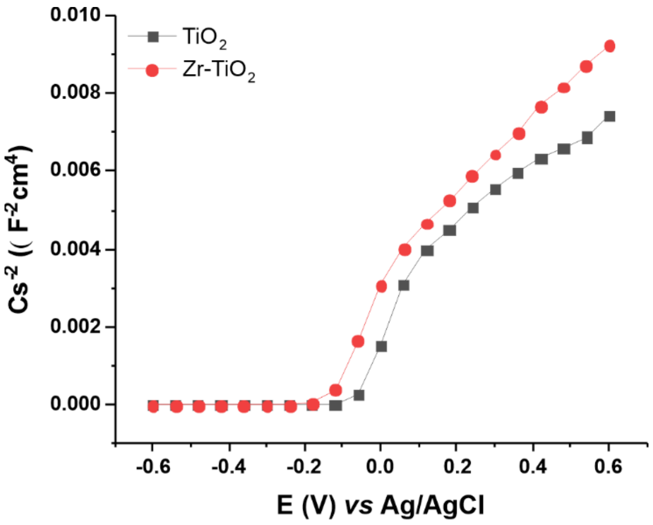


**Figure S28:** Mott-Schottky plot for the films of $\mathbf{TiO_2}$ and **Zr-**$\mathbf{TiO_2}$ (1.00 at% Zr) prepared on FTO coated glass substrate; Pt-mesh counter electrode, Ag/AgCl (3 M NaCl) reference electrode, electrolyte is air saturated 100 mM $HClO_4$ (aq).

**Calculation of Conductivity and Mobility**

$$\sigma_n = \frac{L}{R.A.(1-p)} \qquad (1)$$

where $\sigma_n$ = conductivity, $L$ = thickness of the film, $p$ = porosity of the film, $A$ = geometric area of the electrode

The electronic transport within these nanoporous films is diffusive in nature. Hence, electron mobility can be extracted from the conductivity values using the following equation,

$$\mu = \frac{\sigma_n}{ne} \qquad (2)$$

where $\mu$ = charge carrier mobility, $e$ = electronic charge and $n$ = charge carrier density determined from a Mott-Schottky analysis.

Table S6. Textual measurements used for calculation of conductivity and mobility values presented in Table 1.

| Sample | Sintering Temp. (˚C) | Thickness (μm) | Film area ($mm^2$) | Average porosity (%) |
|---|---|---|---|---|
| **$TiO_2$** | 450 | 7.16 | 30 | 70.3 |
| **$TiO_2$** | 550 | 6.9 | 36 | 70.3 |
| **Zr-$TiO_2$** (1.00 at% Zr) | 450 | 4.42 | 50 | 58.9 |
| **Zr-$TiO_2$** (1.00 at% Zr) | 550 | 4.42 | 50 | 58.9 |
| **Zr-$TiO_2$** (3.15 at% Zr) | 450 | 7.50 | 49 | 65.0 |
| **Zr-$TiO_2$** (3.15 at% Zr) | 550 | 7.50 | 49 | 65.0 |

Table S6. Examples of conductivity and mobility values for $TiO_2$ and method of measurement.

| $TiO_2$ Polymorph | Morphology | Size (nm) | Conductivity ($Scm^{-1}$) | Method of Measurement$^a$ | Mobility ($cm^2V^{-1}s^{-1}$) | Method of Measurement$^a$ | Ref |
|---|---|---|---|---|---|---|---|
| anatase | Spherical | 20 | $3.7 \times 10^{-3}$ | E-FET | $2.3 \times 10^{-4}$ | E-FET | 4 |
| anatase | Irregular | 14.5± 0.5 | ~ $10^{-4}$ | E-FET | - | - | 5 |
| anatase | not described | 16 | - | - | $7 \times 10^{-6}$ | Transient photovoltage, SCLC | 6 |
| rutile | Irregular | 20 | $1.26 \times 10^{-8}$ | I-V | $1.08 \times 10^{-3}$ | SCLC | 7 |
| rutile | Single crystalline nanowire | 10-20 × 1260 | - | - | $1.95 \times 10^{-5}$ | ToF and SCLC | 8 |
| P25 | not described | - | $8.77 \times 10^{-8}$ | I-V | $8.13 \times 10^{-7}$ | SCLC | 7 |
| P25 | not described | - | - | - | $3.6 \times 10^{-2}$ | EIS | 9 |
| not described | Irregular | 49 | ~$10^{-6}$ | EIS | - | - | 10 |
| not described | Irregular | 20 | - | - | 10 | Terahertz Spectroscopy | 11 |
| not described | Irregular | 9 & 300 | - | - | $3.4 \times 10^{-4}$ | SCLC | 12 |

$^a$Abbreviations, Space Charge Limited Current Method (SCLC); Time of Flight measurement (ToF); Electrochemical Impedance Spectroscopy (EIS); Electrochemical Field Effect Transistor Measurement (E-FET)

**References**


(1) Playford, H. Y., Variations in the Local Structure of Nano-Sized Anatase $TiO_2$. *J. Solid State Chem.* **2020,** *288*, 121414.

(2) Di Valentin, C.; Finazzi, E.; Pacchioni, G.; Selloni, A.; Livraghi, S.; Paganini, M. C.; Giamello, E., N-doped $TiO_2$: Theory and Experiment. *Chem. Phys.* **2007,** *339*, 44-56.

(3) Coronado, J. M.; Maira, A. J.; Conesa, J. C.; Yeung, K. L.; Augugliaro, V.; Soria, J., EPR Study of the Surface Characteristics of Nanostructured TiO2 under UV Irradiation. *Langmuir* **2001,** *17*, 5368-5374.

(4) Abayev, I.; Zaban, A.; Fabregat-Santiago, F.; Bisquert, J., Electronic Conductivity in Nanostructured $TiO_2$ Films Permeated with Electrolyte. *Phys. Status Solidi A* **2003,** *196*, R4-R6.

(5) Rettenmaier, K.; Zickler, G. A.; Redhammer, G. J.; Anta, J. A.; Berger, T., Particle Consolidation and Electron Transport in Anatase $TiO_2$ Nanocrystal Films. *ACS Appl. Mater. Interfaces* **2019,** *11*, 39859-39874.

(6) Dittrich, T., Porous $TiO_2$: Electron Transport and Application to Dye Sensitized Injection Solar Cells. *Phys. Status Solidi A* **2000,** *182*, 447-455.

(7) Purkayastha, M. D.; Middya, S.; Datta, J.; Ray, P. P.; Biswas, B. D.; Sarkar, M.; Darbha, G. K.; Singh, N.; Majumder, T. P.; Saha, P.; Das, D., The Carrier Transport Properties and Photodegradation Ability of Low Temperature Synthesized Phase Pure Rutile Titanium Oxide Nanostructured Materials. *Mater. Chem. Phys.* **2019,** *226*, 362-370.

(8) Mohammadpour, A.; Farsinezhad, S.; Wiltshire, B. D.; Shankar, K., Majority Carrier Transport in Single Crystal Rutile Nanowire Arrays. *Phys. Status Solidi RRL* **2014,** *8*, 512-516.

(9) Zhu, T.; Li, C.; Yang, W.; Zhao, X.; Wang, X.; Tang, C.; Mi, B.; Gao, Z.; Huang, W.; Deng, W., Electrospray Dense Suspensions of $TiO_2$ Nanoparticles for Dye Sensitized Solar Cells. *Aerosol Sci. Technol.* **2013,** *47*, 1302-1309.

(10) Fabregat-Santiago, F.; Garcia-Belmonte, G.; Bisquert, J.; Zaban, A.; Salvador, P., Decoupling of Transport, Charge Storage, and Interfacial Charge Transfer in the Nanocrystalline $TiO_2$/Electrolyte System by Impedance Methods. *J. Phys. Chem. B* **2002,** *106*, 334-339.

(11) Tiwana, P.; Docampo, P.; Johnston, M. B.; Snaith, H. J.; Herz, L. M., Electron Mobility and Injection Dynamics in Mesoporous ZnO, $SnO_2$, and $TiO_2$ Films Used in Dye-Sensitized Solar Cells. *ACS Nano* **2011,** *5*, 5158-5166.

(12) O'Hayre, R.; Nanu, M.; Schoonman, J.; Goossens, A., Mott−Schottky and Charge-Transport Analysis of Nanoporous Titanium Dioxide Films in Air. *J. Phys. Chem. C* **2007,** *111*, 4809-4814.
